# Intratumoral heterogeneity and potential treatment strategies in small cell lung cancer

**DOI:** 10.3389/fonc.2025.1657441

**Published:** 2025-10-27

**Authors:** Yunke Yang, Nana Liu, Jiaji Wu, Qingzhe Wu, Jiangnan Zhao, Yue Shi, Mo Shen, Zhiyong Xu, Yueli Shi, Jianghao Yu, Peng Yi, Jin Cheng, Junhui Sun, Yun Xu, Kai Wang

**Affiliations:** ^1^ Department of Respiratory and Critical Care Medicine, Center for Oncology Medicine, the Fourth Affiliated Hospital of School of Medicine, and International School of Medicine, International Institutes of Medicine, Zhejiang University, Yiwu, China; ^2^ Zhejiang Key Laboratory of Precision Diagnosis and Treatment for Lung Cancer, The International Institutes of Medicine, Zhejiang University, Yiwu, China; ^3^ Department of Lung Transplantation, First Affiliated Hospital, School of Medical, Zhejiang University, Hangzhou, China; ^4^ Department of Cardiothoracic Surgery, Center for Oncology Medical, the Fourth Affiliated Hospital of School of Medicine, and International School of Medicine, International Institutes of Medicine, Zhejiang University, Yiwu, China; ^5^ The Quzhou Affiliated Hospital of Wenzhou Medical University, Quzhou People’s Hospital, Quzhou, China; ^6^ The Affiliated Wuxi Center for Disease Control and Prevention of Nanjing Medical University, Wuxi Center for Disease Control and Prevention, Wuxi, China; ^7^ Department of Reproductive Medicine Center, The First Affiliated Hospital of Wenzhou Medical University, Wenzhou, China

**Keywords:** small cell lung cancer, tumor heterogeneity, neuroendocrine, plasticity, ASCL1, neuroD1, tumor microenvironment

## Abstract

Small cell lung cancer (SCLC) is a highly aggressive malignancy characterized by early metastasis and poor prognosis due to the limited efficacy of current treatments. Although initially responsive to chemotherapy and radiotherapy, the majority of patients with SCLC develop resistance within a year, often succumbing to distant metastases. Historically, SCLC was considered a homogeneous disease, primarily driven by the deletion or inactivation of key tumor suppressor genes *TP53* and *RB1*. However, recent advancements in genomics and single-cell sequencing have identified distinct molecular subtypes of SCLC, derived from studies on cell lines, animal models, and tumor tissues. The tumor’s complexity, marked by the coexistence of multiple dynamic subtypes, contributes to its pronounced heterogeneity. Notably, different subpopulations exhibit a complex spatial relationship characterized by both mutual exclusion and coexistence. Temporally, SCLC exhibits the ability to undergo subtype transformations through various molecular mechanisms, underscoring the tumor’s plasticity and offering novel perspectives for personalized treatment approaches. This review synthesizes recent discoveries regarding SCLC subtype classification, intratumor heterogeneity, plasticity-related signaling pathways, immune landscape, and emerging therapeutic strategies.

## 1 Introduction

Lung cancer is among the most prevalent malignancies, representing 12% of newly diagnosed cancer cases globally, and remains the leading cause of cancer-related mortality, placing substantial economic and emotional strain on both society and individuals (1). Lung cancer is broadly categorized into small cell lung cancer (SCLC) and non-small cell lung cancer (NSCLC), with SCLC comprising 15% of cases and NSCLC 85% (2). SCLC is particularly noted for its aggressive nature, rapid progression, high recurrence and metastasis rates, and poor prognosis. Clinically, it is classified as either Extensive-Stage Small Cell Lung Cancer (ES-SCLC) or Limited-Stage Small Cell Lung Cancer (LS-SCLC). The disease predominantly occurs in smokers, with most diagnoses occurring at the metastatic stage (3). While early-stage SCLC responds well to radiotherapy and chemotherapy, relapse is common, and the 5-year overall survival (OS) rate hovers around 10%, with recurrence and drug resistance serving as the primary causes of death (4–6).

Previously, SCLC was regarded as a relatively homogeneous tumor, primarily characterized by the inactivation of key tumor suppressor genes, *TP53* and *RB1* (7). However, recent advancements in genomics and single-cell sequencing have refined the molecular classification of SCLC. Based on the expression of transcription factors such as achaete-scute homolog 1 (ASCL1; also known as ASH-1), neurogenic differentiation factor 1 (NEUROD1), and POU class 2 homeobox 3 (POU2F3), SCLC is now subdivided into four categories: SCLC-A (high ASCL1 expression), SCLC-N (high NEUROD1 expression), SCLC-P (high POU2F3 expression), and SCLC-I (low expression of the three transcription factors but elevated levels of inflammatory markers) (8). SCLC exhibits significant spatiotemporal heterogeneity, marked by dynamic subtype transitions. These variations influence tumor cell growth, invasiveness, drug response, and prognosis. Intratumoral heterogeneity is further complicated by the coexistence and mutual exclusion of subtypes, presenting challenges in treatment. Temporally, subtype shifts may arise due to tumor progression, therapeutic intervention, or external factors. Furthermore, the distinct immune microenvironment of each SCLC subtype may significantly affect immunotherapy outcomes, with the inflammatory subtype responding more favorably to immunotherapeutic approaches. In recent years, immunotherapy has revolutionized SCLC treatment (3, 9, 10), with PD-1/PD-L1 inhibitors, such as Atezolizumab and Durvalumab, showing benefits in first-line treatment of ES-SCLC, extending median OS beyond one year (11, 12) Despite these advancements, current clinical treatment for SCLC largely depends on disease stage and metastasis, with no established therapies specifically targeting the molecular subtypes. Therefore, developing subtype-specific therapeutic strategies and understanding the heterogeneity of SCLC are essential for guiding combination treatments and optimizing intervention timing (13). This review comprehensively examines the latest findings on SCLC subtype classification, intratumor heterogeneity, tumor plasticity, immune microenvironment, and emerging therapeutic strategies.

## 2 Classification of molecular subtypes of SCLC

SCLC was initially regarded as a relatively homogeneous tumor. In 1985, early histological analysis by Carney et al. on 50 SCLC cell lines identified two subtypes: classic and variant. The classic subtype consists of tightly to loosely packed cells, often floating together with or without central necrosis, exhibiting low cloning efficiency in semisolid media, and expressing a full range of SCLC biochemical markers (DDC, BLI, NSE, and CK-BB). In contrast, the variant subtype grows as an adherent monolayer, displaying lower levels of DDC and BLI molecules (14, 15). By 2013, Poirier et al.’s research on Seneca Valley Virus (SVV-001) introduced a novel classification method, demonstrating that the ratio of ASCL1 to NEUROD1 genes could effectively predict SCLC’s response to SVV-001 treatment (16). In 2015, DNA methylation studies categorized SCLC into three clusters: SC-E2, SC-E1, and SQ-P. The SC-E1 subtype exhibited high NEUROD1 and low ASCL1 expression, while SC-E2 showed the reverse, and SQ-P expressed neither, representing a new SCLC subtype (17). That same year, the WHO classified SCLC as a neuroendocrine (NE) tumor, yet some SCLC samples were negative for NE markers, suggesting this definition required further refinement (18, 19). In 2018, Zhang et al. classified SCLC into NE and non-neuroendocrine (non-NE) types based on NE status. NE-type SCLC predominantly expressed transcription factors like ASCL1, NEUROD1, and NKX2-1, while lacking NE inhibitory factors such as REST, and grew as non-adherent floating aggregates or spheres. The non-NE type, characterized by loose adherent cell morphology, primarily expressed POU2F3 and activated pathways such as Notch, Hippo, and TGF-β, with a propensity for undergoing epithelial-mesenchymal transition (EMT) (20). In 2020, Baine et al., employing RNA-seq technology, proposed a four-subtype classification: SCLC-A, SCLC-N, SCLC-P, and SCLC-Y (characterized by high YAP1 expression) (21). However, later sequencing data revealed that YAP1, a transcriptional regulator inhibited by the Hippo signaling pathway, was expressed across SCLC-A, SCLC-P, and SCLC-N subtypes without clear specificity. Immunohistochemical analyses failed to support YAP1-expressing tumors as a distinct subtype, indicating earlier classifications were inaccurate (22).

Subsequent research delved further into refining SCLC classification. In 2021, Gay et al. established a widely accepted four-subtype system based on transcription factors and inflammatory markers: SCLC-A, SCLC-N, SCLC-P, and SCLC-I, which has since become the most commonly used classification method (8). Nonetheless, alternative findings have emerged. In 2019, Wooten et al. identified a previously unrecognized ASCL1+ neuroendocrine variant (NEv2 or SCLC-A2), exhibiting greater resistance to a range of tumor drugs and research compounds (23). More recently, in 2024, Nabet et al. further refined the SCLC-I subtype, distinguishing between SCLC-I-NE and SCLC-I-nonNE, underscoring the increasing complexity of SCLC subtypes (24).

In addition to traditional markers such as ASCL1, NEUROD1, and POU2F3, several studies have identified novel biomarkers for SCLC, including INSM1 and ATOH1. Insulinoma-associated protein 1 (INSM1), a transcription factor from the insulinoma-associated protein family, plays a pivotal role in cell proliferation, differentiation, migration, and neurodevelopment. In SCLC, INSM1 expression is recognized as a highly sensitive and specific nuclear marker of NE differentiation and has been linked to increased sensitivity to chemotherapy agents like irinotecan (25). Atonal Homolog 1 (ATOH1), also known as Mathematician 1 (Math1), is a basic helix-loop-helix (bHLH) transcription factor involved in regulating cell fate and differentiation. In SCLC, ATOH1 is associated with tumor-initiating capacity and NE differentiation, initially identified in SCLC cell line-derived xenografts (CDX) and promoting tumor cell survival and metastasis (26, 27). In 2024, Liu et al. employed multi-omics data to classify 107 SCLC samples based on mRNA, protein, and phosphorylation profiles into four distinct clusters: nmf1, nmf2, nmf3, and nmf4. Cluster nmf1 exhibited the highest NE score, while nmf3 demonstrated the highest mesenchymal marker expression (28). Additionally, that same year, Zhanyu Wang et al. proposed a completely new subtype-H based on the transcription factor Hepatocyte Nuclear Factor 4 Alpha (HNF4A). HNF4A, together with HNF1A and HNF3, often forms a core regulatory circuit to maintain gastroenteropancreatic (GEP) markers, which may be related to the tissue of origin and sensitivity to chemotherapy or targeted therapy. The subtype-H shows a mixed NE phenotype with high Chromogranin A (CHGA) expression and low Neural cell adhesion molecule 1 (NCAM1) expression and exhibits a gastrointestinal-like signature and poor chemotherapeutic response (29). As molecular classification schemes for SCLC continue to evolve, it has become evident that these classifications are correlated with tumor metastasis and drug resistance. Comparative analyses of cell composition and gene expression across subtypes revealed that SCLC-N was more prevalent in lymph node and distant metastases compared to SCLC-A (30). In a multi-omics assessment of 437 metastatic SCLC cases, samples were categorized as A, N, P, Y, or mixed subtypes, with frequencies of 35.7%, 17.6%, 6.4%, 21.1%, and 19.2%, respectively (31). Moreover, activation of the TGF-β signaling pathway in non-NE subtypes has been shown to promote liver metastasis, providing a new avenue for targeted metastasis treatment (31).

**Figure 1:**
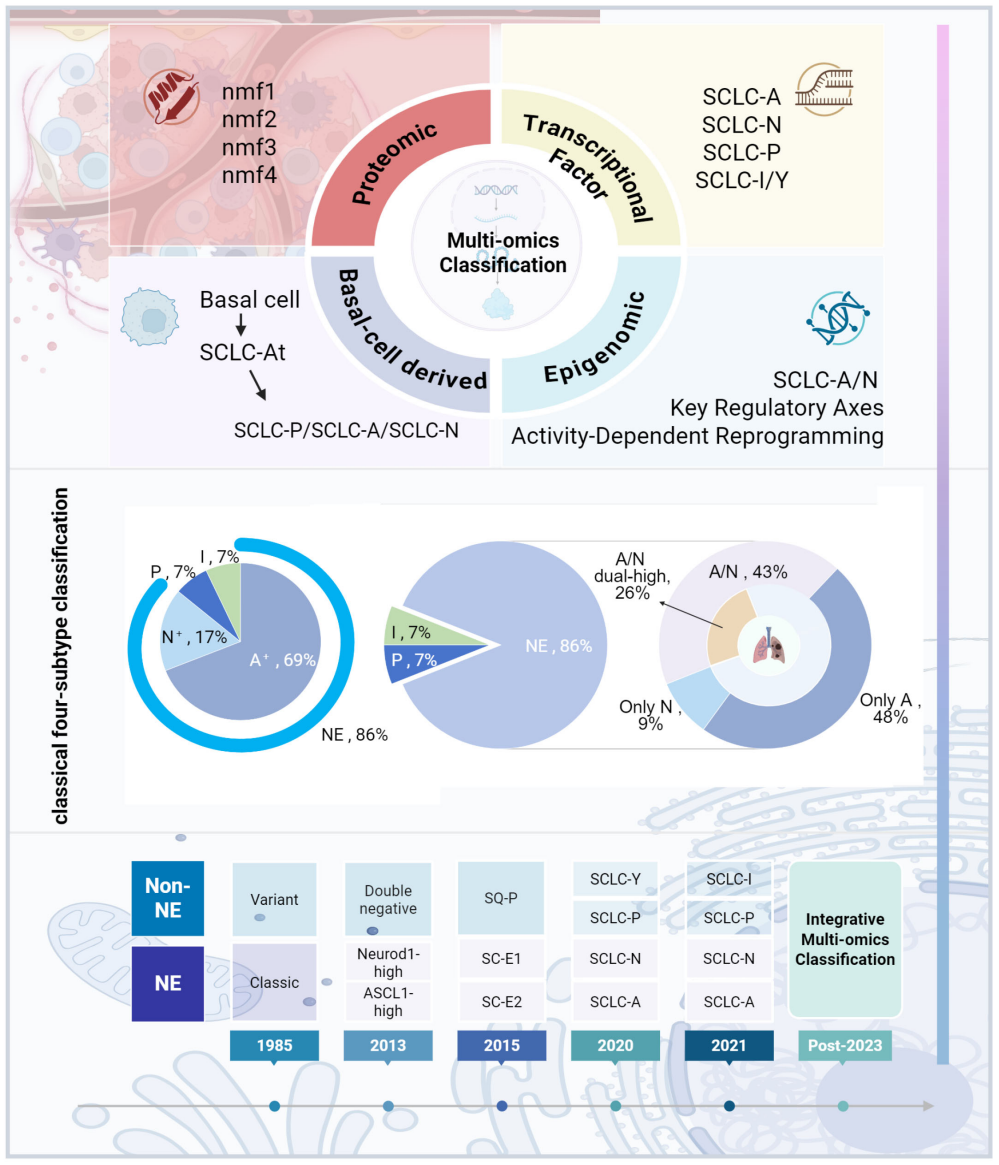
SCLC subtype classification development. The figure was created with BioRender.com.

Chemotherapy has also been found to influence subtype expression. Studies utilizing SCLC CDX and patient-derived xenografts (PDX) models observed an increase in non-NE phenotypes following chemotherapy, accompanied by a decline in NE markers (32, 33). Wagner et al. discovered a significant reduction in ASCL1 expression in chemotherapy-resistant cell lines and post-chemotherapy human tissue samples, suggesting that ASCL1-positive tumor cells are more susceptible to chemotherapy (34). Transcriptome analysis has shown that SCLC-A and SCLC-N subtypes are more responsive to cisplatin, while SCLC-I is the most resistant (32). Furthermore, genomic studies revealed that cisplatin treatment in SCLC-A PDX resulted in tumor progression and metastasis towards the SCLC-I subtype, indicating that subtype conversion may be a mechanism underlying platinum-based drug resistance (8). These findings highlight the clinical relevance of integrating SCLC subtypes with factors such as metastasis and drug resistance. However, the classification of molecular subtypes still requires validation in large-scale studies, and its applicability and accuracy in clinical practice remain to be fully established. The significant heterogeneity within SCLC, driven by tumor evolution, metastasis, and acquired treatment resistance, challenges existing classification systems, which fail to capture the full complexity of the disease. Moving forward, it is imperative to develop a more precise classification framework by leveraging multi-omics technologies (including spatial transcriptomics and single-cell multiome sequencing) in conjunction with clinical efficacy data. This integrated approach is essential to ultimately realize stratified therapy and accurate prognosis prediction for SCLC patients (**Figure 1**).

## 3 Intratumor heterogeneity of SCLC subtype

Intratumor heterogeneity refers to the variations in genotype, phenotype, and molecular characteristics within different regions of the same tumor. This heterogeneity is intricately linked to the tumor microenvironment and spatial structure, influencing factors such as tumor growth, invasiveness, drug sensitivity, and patient outcomes. Recent advances in transcriptomics, spatial proteomics, and spatial metabolomics have introduced innovative methods to study the intratumor heterogeneity of SCLC. This part focuses on the heterogeneity of SCLC subtypes.

### 3.1 Transcriptional factor typing

Current data indicate that nearly all SCLC cells express one or more of the four key transcription factors—ASCL1, NEUROD1, POU2F3, and YAP1—or exhibit inflammatory gene expression characteristic of the SCLC-I subtype (8). ASCL1 and NEUROD1, both members of the bHLH transcription factor family, are central to regulating NE differentiation (35). Historically, studies suggested that ASCL1 and NEUROD1 are independently expressed, rarely showing co-expression (13, 16, 23). However, RNA-seq analysis by Gopal et al. revealed that a single SCLC tumor may express one or more transcription factors, including ASCL1, NEUROD1, and YAP1, underscoring the complexity of intratumor heterogeneity (36). Emerging evidence indicates that co-expression of ASCL1 and NEUROD1 is more common than previously thought, occurring in 20% to 40% of SCLC tumors (21, 26). Zhang et al. found that the number of tumors with dual high expression of ASCL1 and NEUROD1 exceeds those with high NEUROD1 expression alone, suggesting the presence of distinct subgroups within the tumor that exhibit differing biological behaviors and therapeutic responses (20). This further blurs the boundaries of existing SCLC subtype classifications. Baine et al. observed that almost all cases of dual high expression occurred within the same tumor cell population, with only one case showing subclonal regions with differential ASCL1 and NEUROD1 expression (21). Additionally, studies have noted the occurrence of ASCL1 and NEUROD1 co-expression in SCLC samples in a mutually exclusive manner, implying that co-expression may be restricted to spatially distinct regions (8). This intratumor heterogeneity has also been confirmed in SCLC CDX models, where spatially distinct regions of ASCL1- and NEUROD1-positive cells were found in co-expressing tumors (26). Patient tumor samples co-expressing ASCL1 and NEUROD1 suggest that SCLC-A cells are derived from highly dedifferentiated normal NE cells, while SCLC-AN cells, which co-express both transcription factors, exhibit a higher degree of differentiation. This differentiation pattern hints that SCLC-AN could be classified as a distinct subtype (37, 38). Further investigation into SCLC-AN revealed that although ASCL1 and NEUROD1 are occasionally co-expressed at the tissue level, their expression is largely mutually exclusive at the cellular level. Induction of co-expression in SCLC cell lines demonstrated that concurrent expression of these two transcription factors results in reciprocal suppression, leading to impaired cell growth and increased apoptosis (39). A newly identified H subtype frequently co-expresses ASCL1 and NEUROD1, and its drug resistance involves various NADPH metabolic enzymes downstream of the KEAP1-NRF2 pathway, suggesting the existence of a gene-transcript-metabolism reprogramming-mediated resistance pathway. Integrated immunohistochemistry and spatial copy number variation analysis of a rare case of pulmonary neuroendocrine carcinoma confirmed the coexistence of both A and H subtypes, revealing their origin from a common ancestral clone followed by divergent evolutionary trajectories (29). The co-expression and mutual exclusivity patterns observed in intratumor heterogeneity emphasize the need for personalized treatment strategies for patients, tailored to their unique expression profiles.

In contrast, non-NE subtypes are defined by a fundamentally different biology. POU2F3 and YAP1 are key markers of non-NE subtypes in SCLC. POU2F3 is a transcription factor selectively expressed in tuft cells, a rare type of chemosensory cell found in the respiratory tract that responds to external stimuli by releasing bioactive substances to regulate local epithelial and immune cell functions (40). Using CRISPR gene-editing technology, Huang et al. identified POU2F3 as being overexpressed in the low-NE subgroup of SCLC, linking it to the growth of various human cancer cells, including SCLC (40). Further studies confirmed that POU2F3 is primarily expressed in SCLC tumors that are negative for both ASCL1 and NEUROD1, representing 90% (9 out of 10) of cases (21). Single-cell studies by Baine et al. supported this finding, showing that POU2F3 expression in SCLC is mutually exclusive with ASCL1 and NEUROD1 (21). However, Gay et al. found that although less than 1% of cells in patient-derived SCLC CDX models expressed POU2F3, all POU2F3-positive cells co-expressed ASCL1, challenging the exclusivity (8). Additionally, research from the Berns lab suggests that different cells within the respiratory epithelium can give rise to SCLC-like tumors, indicating that the cellular origin of various subtypes may differ (41). The expression profile of SCLC-P closely mirrors that of pulmonary tuft cells, implying a distinct cellular origin compared to other subtypes (13). This distinction could account for its intratumor heterogeneity, although the small sample size of studies on POU2F3 necessitates further research into its heterogeneity.

YAP1, a key regulator of malignant transformation in numerous tumors, plays a critical role in controlling cell proliferation and stem cell growth (42). Its function as a transcription factor defining distinct isoforms in SCLC has been the subject of debate. While YAP1 is expressed across all subtypes, it is predominantly found in tumors negative for both ASCL1 and NEUROD1 (21). Moreover, studies indicate that YAP1 expression in SCLC-Y cell lines, enriched with SMARCA4 mutations, exhibits characteristics of SMARCA4-deficient malignancies rather than traditional SCLC (22). The expression patterns and intratumor heterogeneity of POU2F3 and YAP1 are closely tied to non-NE subtype traits (21). Further investigation into the intratumor heterogeneity of non-NE-specific transcription factors like POU2F3 and YAP1 could provide new insights into therapeutic strategies for targeting these subtypes.

### 3.2 Proteomics

From a proteomics standpoint, direct evidence has emerged elucidating the intratumoral heterogeneity of SCLC. In 2024, Liu et al. conducted an integrative multi-omics analysis of 107 SCLC samples, categorizing SCLC into four distinct clusters (nmf1 to nmf4) based on protein and phosphoprotein profiles. Among these, the nmf1 subtype exhibited the highest neuroendocrine (NE) score, whereas the nmf3 subtype was enriched in mesenchymal markers, systematically revealing fundamentally divergent protein functional states across SCLC subtypes (28). This heterogeneity is further manifested in the spatial tumor microenvironment: Baine et al. demonstrated via multiplex immunofluorescence that within the same tumor, NE subpopulations (e.g., SCLC-A) specifically overexpress proteins such as INSM1 and ASCL1, while adjacent non-NE subpopulations (e.g., SCLC-P) are enriched with POU2F3 and Vimentin. These mutually exclusive protein expression domains define significant spatial intratumoral heterogeneity. Such heterogeneous protein distribution directly impacts therapeutic response. A prominent example is the patchy distribution of the targetable protein DLL3 within tumors, where only a subset of cell subpopulations highly expresses DLL3, while others show low expression. This spatial limitation explains why targeted agents like Rova-T can only eliminate a fraction of tumor cells, ultimately leading to treatment resistance—a phenomenon widely confirmed by immunohistochemistry across numerous preclinical and clinical studies (21).

### 3.3 Spatial multi-omics

Acknowledging the limitations of classical molecular subtyping in predicting clinical outcomes, another study utilized spatial transcriptomics to classify SCLC into three phenotypes based on intratumoral heterogeneity (ITH) levels: high-ITH (h-ITH), medium-ITH (m-ITH), and low-ITH (l-ITH). GO enrichment analysis indicated that the h-ITH phenotype correlates with cell fate and differentiation processes, m-ITH is enriched for immune-related pathways, and l-ITH is associated with cellular stress responses and organogenesis (43). Research employing CODEX and Visium spatial multi-omics technologies demonstrated that tumor regions with a high MPTC signature, characterized by co-expression of ASCL1 and NEUROD1, were linked to poor patient prognosis. Conversely, regions enriched with the MT2 immune niche, defined by aggregates of M1 macrophages, CD8-positive T cells, and NKT cells, were significantly correlated with favorable clinical outcomes. Further analysis confirmed that the proportion of the MT2 niche is a superior prognostic predictor, independent of both tumor mutational burden (TMB) and PD-L1 expression levels (44). Similarly, spatial transcriptome analysis classified SCLC into Epithelial-Resistant (Epi-I) and Epithelial-Sensitive (Epi-II) subtypes. The Epi-II subtype secretes MIF, which educates M2-type myeloid cells. These cells, in turn, release SPP1, activating the PI3K-AKT pathway and driving the transition from the sensitive Epi-II to the highly proliferative and resistant Epi-I subtype. Targeting the MIF/SPP1 axis may block this transition process, offering a new direction for improving patient outcomes (45). The functional specialization and dynamic interactions among these spatial compartments not only provide a profound explanation for the intricate mechanisms underlying intratumoral heterogeneity in SCLC but also elucidate how the local microenvironment dictates the functions of distinct subpopulations. Thereby, these findings offer critical spatial-dimensional insights for precisely locating high-risk tumor regions and optimizing therapeutic strategies.

### 3.4 Integrated epigenomic and transcriptomic

Epigenetic reprogramming and dysregulated molecular networks are central mechanisms amplifying intratumoral heterogeneity and driving therapy resistance in SCLC. Pharmacological inhibition of EZH2, the histone methyltransferase for H3K27me3, has been shown to restore T cell-mediated killing and upregulate MHC class I expression. These findings indicate that EZH2 inhibition enhances tumor immunogenicity and may subsequently improve responses to immune checkpoint inhibitors in SCLC patients (46–48). Additionally, preclinical evidence demonstrates that EZH2 suppression facilitates the loss of the neuroendocrine phenotype, leading to upregulated *SLFN11*—a key factor that induces lethal replication blockade in response to DNA-damaging agents (47). Alternatively, a chromatin accessibility-based framework has been proposed, categorizing SCLC into novel subtypes (SCLC-NE, SCLC-IM, SCLC-SL). Notably, the stem-like SCLC-SL subtype demonstrates a strong correlation with chemotherapy resistance and unfavorable patient outcomes, thereby establishing it as an independent prognostic predictor (49). Another integrative epigenomic and transcriptomic profiling uncovered a NEUROD1-driven regulatory axis in the SCLC-N subtype, involving PDE2A/miR-139-5p as a subtype-specific marker. This study also delineated a cooperative miRNA targeting mechanism directed against genes including NFIB and NOTCH1, which underlies the molecular heterogeneity in SCLC (38). Characterization of super-enhancers, defined by genome-wide histone modifications, serves as a powerful tool for delineating the lineage of unclassified tumors. The study applied this approach and revealed two distinct epigenomic subclusters within the major SCLC-A subtype: SCLC-Aα and SCLC-Aσ. The SCLC-Aα subcluster is characterized by a core regulatory circuitry formed by the super-enhancers of NKX2–1 and SOX1, which function collaboratively to maintain the neuronal lineage state (50). The NE and non-NE dichotomy in SCLC is epigenetically enforced through distinct DNA methylation landscapes. The new classifiers developed based on these profiles—SCLC-DMC (for tissue) and cfDMC (for circulating tumor DNA)—demonstrated perfect concordance with the RNA-seq gold standard. Most importantly, longitudinal tracking via liquid biopsy using cfDMC revealed therapy-driven subtype conversion, such as a shift from SCLC-A to SCLC-I upon disease progression, which was accompanied by promoter methylation alterations in immune-related genes including CXCL12 (51). In addition, EGFR TKI treatment can induce epigenetic reprogramming in EGFR-mutant lung adenocarcinoma (LUAD), facilitating its transformation into SCLC through a process that involves transitional cell states (52). In a separate classification effort, transformed SCLCs (T-SCLCs) have been categorized into a “LUAD-feature retained” subgroup with high NKX2–1 expression and a “LUAD-feature absent” subgroup harboring canonical SCLC genomic features like *TP53/RB1* co-inactivation. These two subtypes exhibit significant differences in their transcriptomes, patient outcomes, and responses to therapy (53).

### 3.5 Others

**Figure 2:**
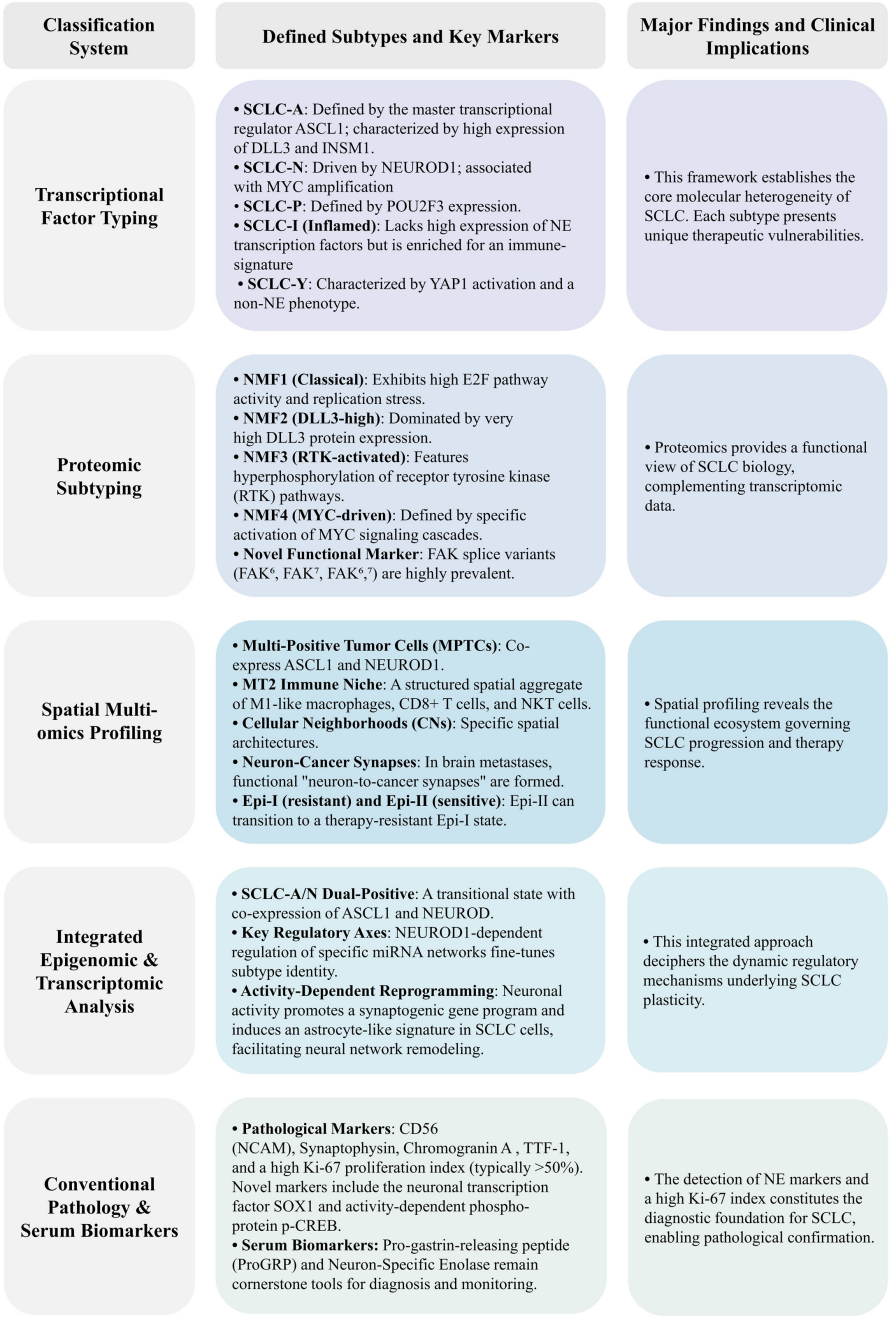
Characterization of SCLC subtypes using different experimental platforms.

Furthermore, intratumoral heterogeneity in SCLC manifests through distinct, coordinated functional layers. At the cellular differentiation level, diffusion pseudotime (DPT) analysis delineated a continuous differentiation spectrum from basal cells to NE or tuft-like cells, namely the “Basal → Atoh1+ → NE/tuft” lineage. Specifically, basal cells first transition into an early Atoh1+ state, which subsequently differentiates further into NE subtypes (SCLC-A/SCLC-N) or the tuft-like subtype (SCLC-P), forming a “trunk-branch” differentiation model. This implies that therapeutic strategies might need to target the differentiation trunk while eliminating the various branched subtypes (54). Furthermore, recent studies highlight electrical activity as a core driver of SCLC malignancy, enhancing metastasis and drug resistance via calcium-dependent signaling pathways (e.g., CREB/FOS). Metabolic heterogeneity, with NE subpopulations relying on OXPHOS and non-NE subpopulations secreting lactate, is key for inter-subpopulation collaboration and also represents a therapeutic vulnerability. Molecules like SOX1, p-CREB, and nAChR show promise as biomarkers for patient stratification towards precision therapy (55). The interplay between tumors and the neural microenvironment is also gaining attention. SCLC cells co-cultured with neurons exhibit enrichment of synapse-related gene signatures, including key genes such as NRXN1, NLGN1, and HOMER1. *In vivo* experiments confirmed that activating cortical neurons (using Thy1-ChR2 transgenic mice) or selectively activating GABAergic interneurons alone (using Dlx-ChRmine transgenic mice) significantly promotes the proliferation of intracranial SCLC, providing a rationale for targeting neuron-tumor interactions (56). Further mechanistic investigation suggests that SCLC hijacks neuronal glutamatergic (and partially GABAergic) synaptic signaling, combined with its intrinsic pulmonary neuroendocrine cell (PNEC)-like phenotype to form synapses, establishing a “neural signaling → SCLC proliferation → neural hyperexcitability” positive feedback loop that exacerbates tumor progression (57). In terms of clinical translational exploration, classification based on TMB indicates that high TMB (TMB-h) is associated with better response to immune checkpoint inhibitors in SCLC (58). Trophoblast cell surface antigen 2 (Trop-2), a cell surface protein implicated in the malignant potential of several cancers including SCLC, has emerged as a potential therapeutic target (59). A phase II study (TROPiCS-03) involving 43 patients with advanced SCLC reported that 41.9% of patients experienced significant tumor shrinkage with a Trop-2-targeting antibody-drug conjugate (ADC), demonstrating promise (60). Other molecular features such as MYC family oncogene amplification, PTEN inactivation, and NOTCH pathway mutations are also implicated in SCLC progression (61). Another study used deep learning to analyze HE-stained whole-slide images, quantified SCLC intratumoral heterogeneity by identifying its histomorphological phenotypes (e.g., HIPO subtypes, HPCs), classified SCLC into subtypes with significant prognostic differences (e.g., HIPOS-I, HIPOS-II) to achieve heterogeneity-based precision prognostic assessment (62). However, biomarker-driven classification systems currently face challenges. Firstly, the high heterogeneity of SCLC limits the utility of single biomarkers for accurate classification (63). Secondly, the co-expression or mutual exclusivity patterns of various biomarkers suggest their expression may vary across different tumor regions. Looking forward, future research should focus on elucidating the driving mechanisms behind the dynamic evolution of SCLC subtypes, understanding the impact of spatial architecture on functional states, and developing multi-target combination or sequential strategies to overcome resistance challenges posed by subtype switching. Constructing dynamic subtyping systems integrating multi-omics data and artificial intelligence algorithms will be crucial for achieving precision therapy in SCLC (**Figure 2**).

## 4 Signaling pathways associated with plasticity and temporal evolution of SCLC subtypes

SCLC genomes can evolve rapidly during treatment, driving increased tumor heterogeneity and subtype transitions, which can impact therapeutic responses (8, 64). Understanding the plasticity and temporal evolution of SCLC is essential for formulating effective treatment strategies. Single-cell RNA-seq has demonstrated that individual SCLC cells can gradually shift between transcription factor subtypes (65). For example, MYC can induce dedifferentiation by enhancing Notch/REST activity, leading to transitions from ASCL1+ to NEUROD1+ and eventually to YAP1+, exemplifying the potential for SCLC subtype transitions (66). Furthermore, clinical data suggest that patients with late-stage SCLC-Y exhibit the highest survival rates, followed by SCLC-P, while the other subtypes show lower survival scores. This implies that molecular mechanisms, such as those involving MYC and Notch, may enable transitions into the SCLC-Y subtype, potentially extending patient survival (67). This part highlights key signaling molecules involved in the plasticity of SCLC, aiming to provide new directions for treatment strategies.

### 4.1 MYC family

The MYC family of genes, including MYC, MYCN, and MYCL, serves as key oncogenic drivers in various tumors. These MYC family proteins, classified as bHLH transcription factors, bind to E-box DNA sequences (CACGTG) and form heterodimers with smaller bHLH proteins like MAX to regulate target gene expression (68). In SCLC, overactivation of MYC family transcription factors influences cell proliferation, controls the cell cycle, and facilitates malignant transformation (69). Notably, the overexpression of MYCN or MYCL in chemotherapy-sensitive PDX models drives the development of chemoresistance in SCLC (70). Studies have demonstrated that MYCL is amplified or highly expressed in the SCLC-A subtype and plays a pivotal role in ASCL1 function, while other subtypes tend to display MYC amplification or overexpression, suggesting that MYC predominates in SCLC with low NE status (13, 69).

Research by Ireland et al. using SCLC genetically engineered mouse models (GEMM) revealed that MYC activates Notch/REST signaling, prompting the transition of SCLC from an ASCL1+ to a NEUROD1+ and eventually to a YAP1+ state. This transformation results in histological features resembling large-cell neuroendocrine carcinoma (LCNEC) (66). Similar patterns were observed in human SCLC cell lines H1963 and H187, providing valuable insights for the evaluation of clinical strategies (71). Ireland et al. demonstrated that loss of ASCL1 (RPMA model) significantly suppresses the neuroendocrine subtype and induces a transition toward SCLC-P and SCLC-Y subtypes. In contrast, PTEN loss (RPP model) activates the PI3K/AKT pathway and upregulates MYC, doubling the proportion of POU2F3+ cells in tumors of basal origin, thereby establishing a molecular mechanism for the cooperative regulation of SCLC-P (54). Additionally, MYC-driven SCLC has shown heightened sensitivity to Aurora kinase inhibitors—serine/threonine protein kinases that, when overexpressed, promote tumor cell proliferation and survival in SCLC (72, 73). Treatment of MYC-driven SCLC mice with the Aurora kinase inhibitor Alisertib, in combination with chemotherapy, significantly extended median survival time compared to controls (74). These findings suggest that MYC family members play a pivotal role in treatment sensitivity in SCLC, positioning them as critical biomarkers for patient stratification and potential targets for therapy (75).

### 4.2 Notch signaling pathway

The mammalian Notch signaling pathway comprises four Notch receptors and five DSL family ligands, interacting with multiple proteins (76). This pathway regulates key physiological functions, including cell proliferation, differentiation, organ development, and tissue homeostasis. Emerging evidence indicates that the Notch signaling pathway plays a pivotal role in the transformation between NE and non-NE characteristics in SCLC, potentially influencing 10% to 50% of tumor cells, contingent on the activation threshold required (65, 77). Target genes of the Notch pathway, such as HES1 and HEY1, are known to suppress ASCL1, a key regulator of NE differentiation in SCLC, thereby facilitating the transition toward a non-NE phenotype (78).

In human SCLC cell line H1688, activation of Notch1 resulted in a reduction of NE marker expression and induced a morphological shift to a glandular cell arrangement, with loosely clustered aggregates indicative of a more non-NE state (79). Both *in vivo* and *in vitro* studies suggest that, alongside MYC, factors like YAP1 can promote REST gene expression *via* a Notch-dependent mechanism, further driving the shift from NE to non-NE characteristics (80). The Notch pathway also has implications in SCLC immunotherapy. Transcriptomic analysis of primary SCLC tumors, patient-derived CDX models, and cell lines revealed that Notch activation upregulates MHC-I gene expression and enhances immune cell infiltration, rendering non-NE subgroups more susceptible to immunotherapy (81). Additionally, Notch1 loss is negatively correlated with PD-L1 expression and can drive the polarization of macrophages toward the M1 type. This reduction in NE differentiation corresponds with an increase in tumor-intrinsic immunity, suggesting a link between Notch signaling activation, the NE differentiation state, and the tumor immune microenvironment (82, 83). A more comprehensive understanding of the Notch pathway and its interplay with other signaling cascades may provide valuable insights for developing targeted therapies that leverage Notch-dependent mechanisms in specific SCLC subtypes.

### 4.3 EZH2

Enhancer of Zeste Homolog 2 (EZH2), the catalytic subunit of Polycomb Repressive Complex 2 (PRC2), induces transcriptional silencing through tri-methylation of histone H3 at lysine 27 (H3K27me3), playing a critical role in cisplatin resistance in SCLC (84). Studies on samples from patients with SCLC, GEMMs, and human cell lines have shown that transient inhibition of EZH2 promotes a transition of SCLC cells from a high-NE state to an inflammatory low-NE state. EZH2 inhibition unblocks the TAP1 gene, a key player in MHC I antigen processing, and activates the STING pathway, a critical component of innate immune signaling^71^. This activation enhances the immune microenvironment by inducing cytokine production, such as interferon, thereby improving tumor immunogenicity. Specifically, EZH2 inhibition upregulates TAP1, restores MHC I expression on the tumor cell surface, and increases the presentation of antigenic peptides, making the tumor more likely to be recognized and targeted by the immune system (47). Moreover, immunohistochemical analysis revealed strong nuclear expression of EZH2 in all SCLC cells. In human SCLC cell lines (H146, H345), EZH2 silences the TGF-β type II receptor (TβRII) *via* epigenetic mechanisms, thereby inhibiting TGF-β-mediated apoptosis. Since TGF-β downregulates ASCL1 through the SMAD pathway, EZH2 promotes SCLC progression by blocking the TGF-β-SMAD-ASCL1 axis, suggesting that reducing EZH2 expression indirectly lowers ASCL1 levels (85). Additionally, EZH2 inhibition has been shown to activate p63 expression, facilitating the transdifferentiation of esophageal neuroendocrine carcinoma cells into squamous cell carcinoma, which improves drug sensitivity and enhances survival rates (86). However, in medulloblastoma (MB), EZH2 inhibition upregulated NEUROD1 and promoted cellular differentiation, highlighting the complexity of EZH2’s role in various cancer contexts (87). This evidence opens the possibility that EZH2 inhibition may facilitate subtype switching in SCLC, potentially influencing therapeutic outcomes. In PDX models, the addition of EZH2 inhibitors to standard chemotherapy restored the expression of key genes, such as *SLFN11*, which are silenced under chemotherapy pressure *via* EZH2-mediated methylation. This reactivation sensitizes tumor cells to chemotherapeutic agents, potentially delaying the development of acquired resistance and improving treatment efficacy (48). Several clinical trials are currently investigating EZH2 inhibitors in advanced solid tumors, including SCLC, such as a phase I trial of Mevrometostat (NCT03460977) and a phase II trial of XNW5004 (NCT06022757), though results are still pending (88). The potential of EZH2 inhibitors in overcoming cisplatin resistance and promoting subtype switching in SCLC presents a promising therapeutic avenue, offering a novel strategy for managing this aggressive cancer.

### 4.4 LSD1

Lysine-specific demethylase 1 (LSD1), encoded by the *KDM1A* gene, is a lysine demethylase intricately linked to malignant transformation, EMT, cell proliferation, and differentiation across various cancers, making it a critical target for cancer therapies (89). LSD1 can bind to Notch sites, suppressing Notch1 expression and its downstream signaling, thus facilitating subtype transitions through the Notch pathway, which significantly influences SCLC development and progression (90). Inhibition of LSD1 in four human SCLC cell lines (H510A, H1417, H146, and H187) using ladademstat, followed by RNA-seq analysis, revealed activation of Notch signaling and upregulation of REST, leading to decreased expression of ASCL1 and other NE lineage genes (90). Another LSD1 inhibitor, T-3775440, was found to reduce NE markers such as CHGA and GRP in SCLC cell lines H1417 and H510A by directly disrupting the interaction between LSD1 and INSM1/GFI1B, leading to the downregulation of ASCL1 expression (91). Additionally, LSD1 can inhibit the key target gene ZFP36L1, which plays a tumor-suppressive role by regulating hypoxia and cell cycle signaling. ZFP36L1 is an mRNA-binding protein that targets AUUUA/UUAUUUAUU elements in the 3’ UTR of genes, leading to mRNA degradation (92, 93). While ZFP36L1 is typically expressed at low levels in the SCLC-A subtype, it is more highly expressed in the inflammatory subtype, suggesting that restoring ZFP36L1 expression could enhance the plasticity of the inflammatory subtype and inhibit NE differentiation and cell proliferation, offering a promising therapeutic avenue (94). Further research is needed to explore the roles of LSD1 and ZFP36L1 in SCLC-N and SCLC-P subtypes. LSD1 and MYC both activate the Notch pathway and promote NE differentiation in SCLC cells, and they are known to interact. Experimental studies have demonstrated that MYC recruits LSD1 to regulate chromatin and DNA oxidation, while LSD1 activity influences MYC-driven transcription, underscoring the importance of their interaction for MYC-mediated gene expression (95). Although no direct evidence currently indicates an upstream-downstream relationship between LSD1 and MYC in the Notch pathway, the possibility that they jointly activate Notch signaling to drive the transition from ASCL1 to NEUROD1 remains to be investigated. These insights suggest that LSD1 inhibitors, by inducing phenotype transitions, may boost SCLC immunogenicity and offer a novel therapeutic approach.

### 4.5 EMT

EMT refers to the process by which epithelial cells lose their defining traits and acquire mesenchymal characteristics, a key mechanism in tumor metastasis, therapy resistance, drug resistance, and embryonic development (96). Single-sample gene set enrichment analysis (ssGSEA) of human SCLC cell lines, RPM mouse tumors, and SCLC CDX tumor samples revealed that the SCLC-A2 subtype is notably enriched with epithelial signature genes, while SCLC-A and SCLC-N display more mesenchymal traits. Other subtypes, such as SCLC-P and SCLC-Y, also tend to exhibit increased mesenchymal features (97). EMT scoring in SCLC samples by Gay et al. further confirmed that the SCLC-A subtype is the most epithelial, while SCLC-I shows the most mesenchymal characteristics, with the remaining subtypes falling between these extremes (8). These observations underscore a strong link between EMT status and SCLC subtype plasticity. EMT is regulated by several signaling pathways within the tumor microenvironment, including TGF-β and Wnt signaling. TGF-β can induce mesenchymal markers like N-cadherin and vimentin while suppressing epithelial markers such as E-cadherin (98). The Wnt pathway, by activating β-catenin and other transcription factors, promotes EMT-related gene expression (99). These pathways interact, facilitating both EMT and SCLC subtype transitions. TGF-β, through SMAD-dependent and SMAD-independent pathways, promotes EMT across multiple cancer types. In human SCLC cell lines H146 and H345, TGF-β downregulates ASCL1 *via* a SMAD-dependent mechanism, suggesting its potential as a target for influencing SCLC phenotype transitions (85, 100). Additionally, research on SCLC cell lines identified that Wnt11, acting as a Wnt ligand, modulates E-cadherin expression and the NE switch in an ASCL1-dependent manner (101). The reversible transition between NE and non-NE cells in SCLC may be driven by EMT mechanisms, which are influenced by dynamic extracellular signals or intracellular factors, offering new therapeutic perspectives for SCLC (97).

### 4.6 SOX2

SRY-Box 2 (SOX2), a key transcription factor for maintaining stem cell pluripotency, plays a significant role in sustaining cell self-renewal and proliferation. Its dysregulated expression is strongly linked to SCLC development, differentiation, metastasis, and poor prognosis (102, 103). Studies have demonstrated that knocking down SOX2 in human SCLC cell lines H69 and H889 inhibits the proliferation of SCLC-A subtype cells and significantly reduces the expression of key molecules associated with the SCLC phenotype, such as INSM1 (104). In the SCLC-N subtype cell lines H29 and H82, SOX2 was found to directly bind to the promoter region of NEUROD1, with overexpression leading to NEUROD1 silencing (105). These findings suggest that inhibiting SOX2 could drive a phenotypic transition from the SCLC-A to the SCLC-N subtype. Further research on the human SCLC cell line CORL47 revealed that ZFP36L1 binds to and downregulates mRNA levels of both SOX2 and INSM1, thereby blocking ASCL1-driven NE differentiation and proliferation (94). Several studies indicate that SOX2 influences the transitions between SCLC subtypes by modulating the expression of ASCL1 and NEUROD1, contributing to tumor heterogeneity (94, 106). Consequently, targeting SOX2 may represent a promising therapeutic strategy for patients with SCLC.

### 4.7 KDM6A

Lysine-specific demethylase 6A (KDM6A) is a histone demethylase that removes di- and tri-methylation from lysine 27 on histone H3 (H3K27), playing a key role in regulating gene expression, cell fate determination, and development (107, 108). Studies have shown that KDM6A inactivation facilitates the transition from the SCLC-A subtype to the SCLC-N subtype. In KDM6A-knockout SCLC GEMMs, KDM6A was found to sustain active chromatin states in SCLC-A through its demethylase activity and scaffolding role in the COMPASS complex. Loss of KDM6A leads to decreased H3K4me1 and increased H3K27me3, thereby activating NE gene enhancers and upregulating NEUROD1 and its target gene PAX6, which promotes the transition from ASCL1 to NEUROD1 (109–111). While KDM6A does not directly upregulate NEUROD1, studies in KDM6A-mutant SCLC GEMMs and cells suggest that other epigenetic modifiers and transcription factors, such as KMT2A and MYC, may interact with KDM6A to increase NEUROD1 expression (111). Ongoing research aims to clarify the mechanisms underlying SCLC subtype transitions, which will guide the development of targeted therapies for specific SCLC subtypes.

### 4.8 KDM5A

**Figure 3:**
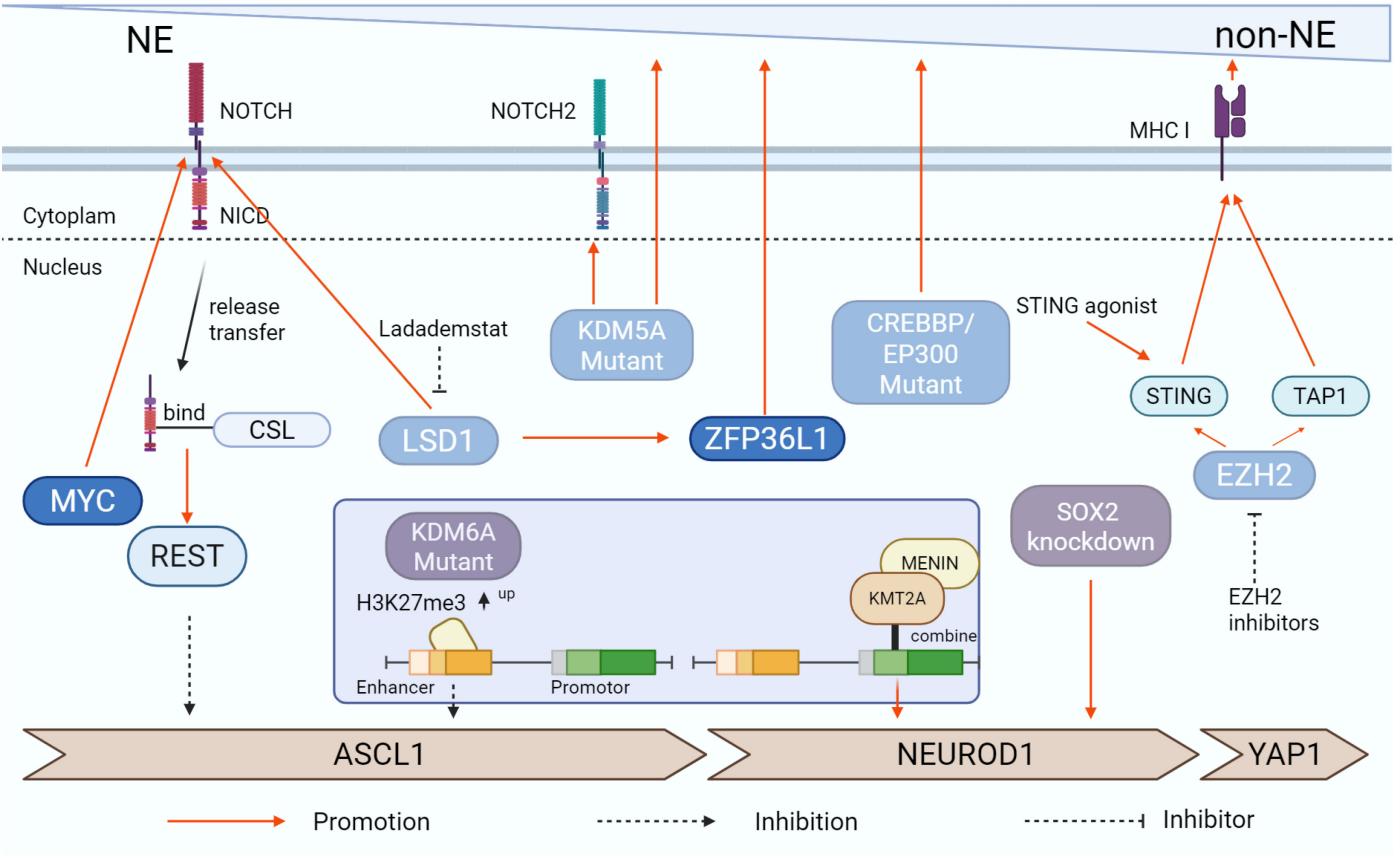
Signaling pathways associated with plasticity and temporal evolution of SCLC subtypes. The study found that diverse signaling pathways are capable of facilitating the phenotypic transition of SCLC. MYC, Notch signaling pathway, and ZFP36L1 are among the factors that can enhance SCLC’s NE differentiation, whereas EZH2 and LSD1 tend to suppress this process. Additionally, KDM6A- and KDM5A-Mutant exert significant influence on these changes. The figure was created with BioRender.com.

KDM5A, also known as JARID1A or RBP2, is a member of the KDM5 family, which removes di- and tri-methylation marks on lysine 4 of histone H3 (H3K4me2/3). This family is closely linked to tumor resistance, EMT, and other critical processes (112). Studies using LSL-Cas9 SCLC GEMMs revealed that KDM5A demethylates H3K4me3 to regulate gene expression and inhibit Notch2 and its downstream targets, which are essential for maintaining high ASCL1 levels and NE differentiation. Inactivation of KDM5A promotes non-NE differentiation in SCLC (113). Although some mechanisms underlying KDM5A’s role in SCLC progression have been uncovered, the precise relationship between KDM5A and different SCLC subtypes remains unclear. Future research will focus on whether KDM5A promotes the transition from SCLC-A to SCLC-N, potentially offering therapeutic insights for modulating SCLC subtypes (**Figure 3**).

Furthermore, several molecules play critical roles in the temporal heterogeneity and plasticity of SCLC subtypes. In SCLC cells, co-culturing with fibroblasts activates the JAK2/STAT3 signaling pathway, which is associated with phenotypic reprogramming (114). Genetic studies using SCLC GEMMs identified TAZ as a pivotal molecular switch that coordinates phenotype transformation and metastasis. The SWI/SNF complex promotes the transition of SCLC cells from non-metastatic to metastatic states through the epigenetic silencing of TAZ (115). VGF promotes the upregulation of the transcription factor ASCL1 through the CREB-dependent pathway, thereby driving neuroendocrine differentiation, and plays a critical role particularly in ASCL1-positive subtypes (116). In SCLC GEMMs and their cells, YAP has been shown to induce REST expression *via* both Notch-dependent and Notch-independent pathways, facilitating the transformation from NE to non-NE cells (80). In the SCLC cell line H69, CSF2 was found to regulate phenotypic plasticity by phosphorylating the STAT3/MYC pathway, restricting the transition between adherent (H69A) and suspension (H69S) phenotypes and altering drug sensitivity in specific cell clones (117). Additionally, the loss of histone acetyltransferases, including CREBBP (also known as CBP) and its paralog EP300 (also known as p300), leads to a reduction in NE-related epithelial markers and a corresponding rise in non-NE markers, such as ZEB1 and VIM, in SCLC GEMM models (118). Mutations in CREBBP/EP300 have been linked to the activation of Notch signaling by regulating FBXW7 in B-cell lymphoma, though the specific mechanism in SCLC remains unclear (119). These molecules present promising therapeutic targets for SCLC.

While studies confirm that SCLC can transition from an NE to a non-NE phenotype and that subtypes can switch between one another, the precise mechanisms underlying these transformations and their impact on drug resistance remain to be fully elucidated. Exploring how the plasticity mechanisms can be harnessed to shift SCLC subtypes toward SCLC-I, which is associated with longer survival, represents a valuable future research direction.

## 5 Immune microenvironment

**Figure 4:**
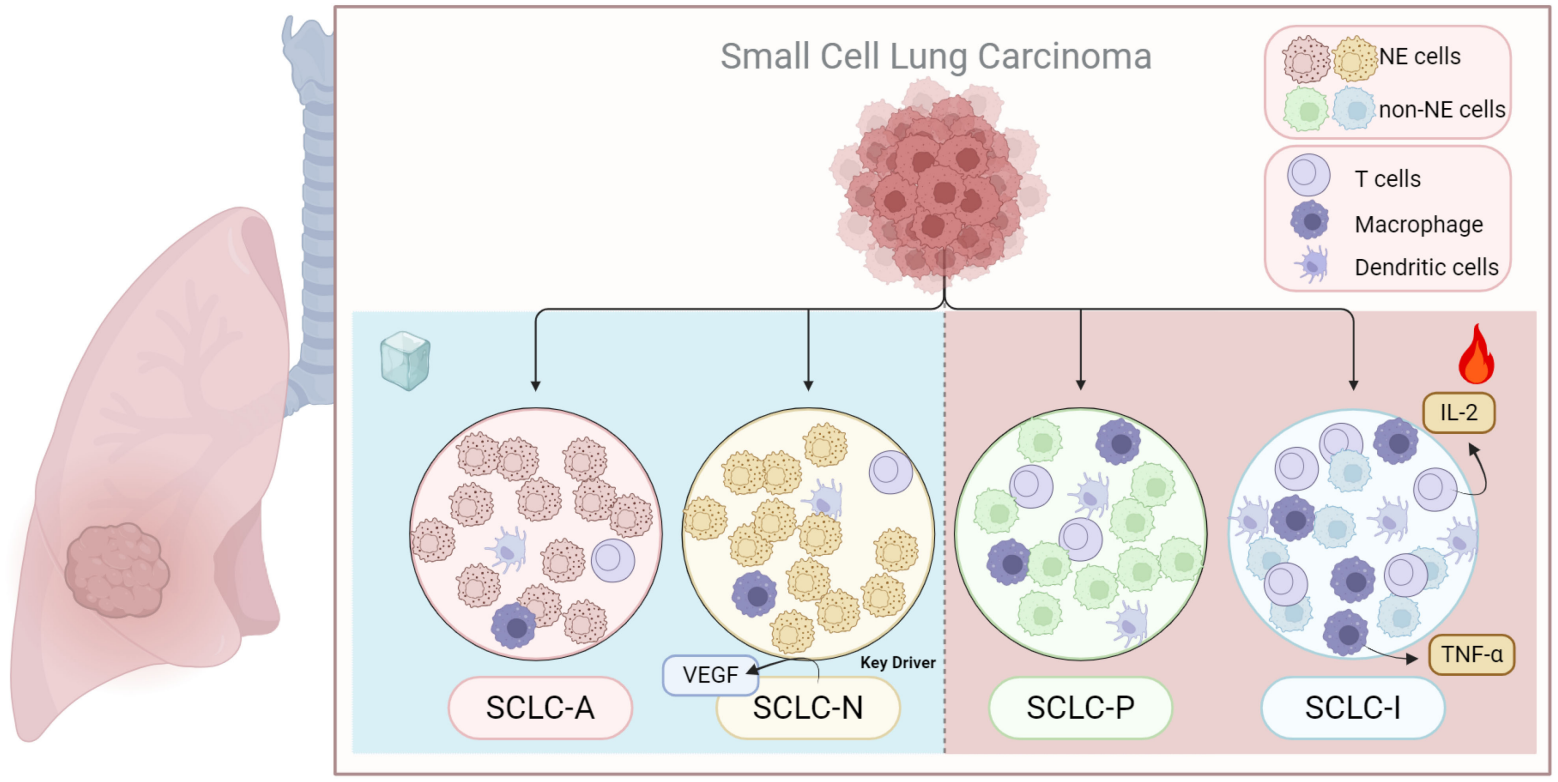
Immunoinfiltration of different subtypes of SCLC. SCLC transcriptional subtypes display distinct immunogenic profiles that may impact response to immunetherapy. The SCLC-I tend to P has a higher immunological profile, while SCLC-N and SCLC-A has been suggested to be the most immune-cold. The figure was created with BioRender.com.

The immune microenvironment in SCLC is largely immunosuppressive, characterized by an accumulation of regulatory T cells (Tregs) and myeloid-derived suppressor cells (MDSCs), along with low levels of T-cell infiltration, which contributes to poor responsiveness to immune checkpoint inhibitor (ICI) therapies (120, 121) (**Figure 4**). Different SCLC subtypes are associated with distinct tumor microenvironments, which lead to varying degrees of immune resistance and responses (122). Research by Chan et al. revealed that the ratio of CD8+ effector cells to Tregs in the SCLC-N subtype is significantly lower than in SCLC-A, correlating with a poorer prognosis for patients with SCLC-N (30). Another study found that the SCLC-P subtype had a higher absolute abundance of CD8+ T cells compared to SCLC-A and SCLC-N (123). Among the subtypes, SCLC-A tumors displayed higher expression of immunosuppressive receptors (FoxP3, PD2, and CTLA4) and lower levels of immune-promoting receptors (CD8), whereas SCLC-I tumors exhibited the highest immune cell infiltration, with notably increased T cells, NK cells, and macrophages (8, 124). In the Impower133 trial, patients with the EMT-like SCLC-I subtype showed significantly improved OS when treated with a combination of chemotherapy and the PD-L1 monoclonal antibody atezolizumab, compared to other subtypes (8). This suggests that targeting subtype conversion could be a viable treatment approach. The spatial distribution of immune cells in SCLC also contributes to immune evasion and resistance to immunotherapy. Cytotoxic T lymphocytes (CTLs), which are rare in SCLC, are found at much higher densities in the stroma compared to the tumor parenchyma (125). In PD-1-positive SCLC samples, tumor-infiltrating lymphocytes (TILs) are typically located at the tumor-stroma interface, with limited presence among cancer cells, although more CD8+ T cells are observed at the tumor margins (122, 126). It also indicates that tumor cells might actively exclude cytotoxic T cells by creating physical or chemical barriers, hindering their entry into the tumor nests to mediate cytotoxicity. Factors driving SCLC subtype transitions also impact the immune microenvironment. For instance, EZH2 inhibition in patient samples, mouse models, and human SCLC cell lines activates the PRC2-mediated MHC-I antigen processing pathway, restoring T-cell-mediated tumor immunity (46). RNA-seq analysis of the human SCLC cell line CORL47 revealed high expression of HLA-B and HLA-C in ZFP36L1-activated SCLC, indicating an increased number of antigenic peptides on the tumor cell surface, making these tumors potentially more sensitive to T-cell-based immunotherapies (94). Understanding the immune landscape in various SCLC subtypes is essential for designing effective immunotherapy strategies and optimizing treatment timing (**Figure 4**).

## 6 Precision treatment strategies for different SCLC subtypes

Currently, all patients with SCLC are treated with platinum-based first-line chemotherapy, radiation therapy, and immunotherapy. However, advancements in molecular subtyping and gene-level insights present opportunities for new therapeutic approaches tailored to specific SCLC subtypes. By identifying key signaling pathways and drug resistance mechanism within these subtypes, more targeted and potentially effective treatment strategies can be developed (127).

### 6.1 SCLC-A

SCLC-A, the most common subtype, accounts for 40-50% of all SCLC cases. Cells in this subtype exhibit classic morphology and high expression of NE markers such as CD56 and CHGA (21). Key molecular features of SCLC-A include elevated levels of Delta-Like Ligand 3 (DLL3) and INSM1, while CREBBP expression is reduced (8). Potential targets for this subtype include DLL3, a protein downstream of the transcription factor ASCL1, as well as B-cell lymphoma 2 (BCL2) and LSD1, both of which are involved in histone modification. DLL3-targeted therapies, such as antibody-drug conjugates, bispecific T-cell engagers, and chimeric antigen receptor (CAR) T-cell constructs, are currently under clinical development. As DLL3 expression correlates with ASCL1, SCLC-A may be particularly sensitive to DLL3-targeted therapies (128). Tarlatamab showed promising Phase I results, with a disease control rate (DCR) of over 50%, significantly improving patient survival (129). Inhibition of BCL2 has also been effective in controlling SCLC growth in both *in vitro* and *in vivo* models (8, 130, 131). Additionally, inhibiting LSD1 activates Notch signaling and suppresses ASCL1 expression. The LSD1 inhibitor Bomedemstat improved SCLC response to PD-1 inhibitors in mouse models, and ongoing clinical trials are evaluating its efficacy (NCT05191797) (90, 132). Another potential therapeutic target in SCLC-A is the CDK2-CyclinA2 complex, which phosphorylates ASCL1 and promotes its degradation during mitosis, suggesting a new therapeutic avenue (133). In summary, DLL3, BCL2, and LSD1 represent promising targets for treating SCLC-A.

### 6.2 SCLC-N

SCLC-N is primarily characterized by the expression of NEUROD1 and NE markers such as synaptophysin, chromogranin A, and CD56/NCAM (8). MYC amplification is frequently associated with the this subtype and represents a key mechanism imparting therapeutic resistance in SCLC (134–137), making it more responsive to Aurora kinase inhibitors, CHK1 inhibitors, and other targeted therapies (74, 138–140). Short-term follow-up findings from a phase II trial demonstrated that combining Aurora kinase inhibitors with chemotherapy improved progression-free survival (PFS) and OS (134). SCLC-N also relies on arginine metabolism. A key mechanism find that chronic exposure to ADI-PEG 20 can induce the reexpression of argininosuccinate synthase (ASS1) in tumor cells, thereby restoring endogenous arginine biosynthesis and conferring acquired resistance to arginine deprivation therapy (141, 142). With *in vitro* studies showing that arginine deiminase (ADI) and arginase (ARG) exhibit cytotoxicity in SCLC cell lines, particularly those lacking argininosuccinate synthase (ASS) (136, 141, 143, 144). Clinical trials are ongoing to evaluate the efficacy of these metabolic inhibitors (NCT05616624). Moreover, SCLC-N shows sensitivity to the SVV oncolytic virus, and combining SVV with immunotherapy holds promise for enhanced therapeutic outcomes (16, 145). Blocking transcriptional co-activator BET proteins also reduces NEUROD1 expression, thereby inhibiting SCLC growth both *in vitro* and *in vivo* (146). Furthermore, KRS1, a kinase inhibitor of RAS1, has been identified as a driver of cisplatin resistance and may represent a therapeutic target in SCLC-N (147). These findings highlight the potential of therapies targeting Aurora kinases, arginine metabolism, and SVV oncolytic virus for more effective treatment of SCLC-N.

### 6.3 SCLC-P

SCLC-P predominantly expresses POU2F3 with low or absent NE marker expression. CRISPR screening has revealed that the SCLC-P subtype uniquely depends on Insulin-like Growth Factor 1 Receptor (IGF-1R), suggesting that linsitinib, an IGF-1R inhibitor, could be a potential therapeutic option for these patients (40). However, a phase II clinical study of OSI-906 (an oral tyrosine kinase inhibitor [TKI] of IGF-1R) combined with topotecan in relapsed SCLC demonstrated safety but limited clinical activity, indicating the need for further investigation (148). Given the frequent loss of *TP53* and *RB1* in SCLC, these tumors are particularly vulnerable to DNA damage. Targeting DNA damage response mechanisms, such as Poly ADP-ribose polymerase (PARP) and RAD3-related protein (ATR), has shown promise in patients with SCLC-P (8, 149). PARP inhibitors, which disrupt DNA repair mechanisms, induce genomic instability and cell cycle arrest, exhibiting anti-tumor effects *in vitro* and *in vivo* (150, 151). Adding the PARP inhibitor veliparib to cisplatin and etoposide in first-line chemotherapy for ES-SCLC resulted in improved objective response rate (ORR), PFS, and OS (75). In addition, a phase II trial evaluating olaparib (a PARP inhibitor) with ceralasertib (an ATR inhibitor) in relapsed or refractory SCLC showed promising disease stabilization, although it did not meet predefined endpoints (152). *In vitro* studies have also demonstrated that SCLC-P is particularly sensitive to antimetabolites, including antifolates and nucleoside analogs (8, 153). Additionally, the marine-derived anticancer agent lurbinectedin, an inhibitor of RNA polymerase II, selectively disrupts oncogene transcription processes and has shown the highest efficacy in the SCLC-P subtype, particularly in the human SCLC cell line H526. A phase I trial conducted in China reported an overall response rate of 45.5% for lurbinectedin as a second-line treatment (154, 155). The PTEN-MYC axis drives SCLC-P pathogenesis, revealing a rationale for PI3K/AKT inhibition (54). Concurrently, mSWI/SNF complex activity (SMARCA4/2) is essential for the POU2F3 transcriptional program, and its loss desensitizes this subtype to corresponding inhibitors (156). These findings suggest that PARP inhibitors and IGF-1R inhibitors may offer promising therapeutic options for patients with SCLC-P.

### 6.4 SCLC-I/Y

**Figure 5:**
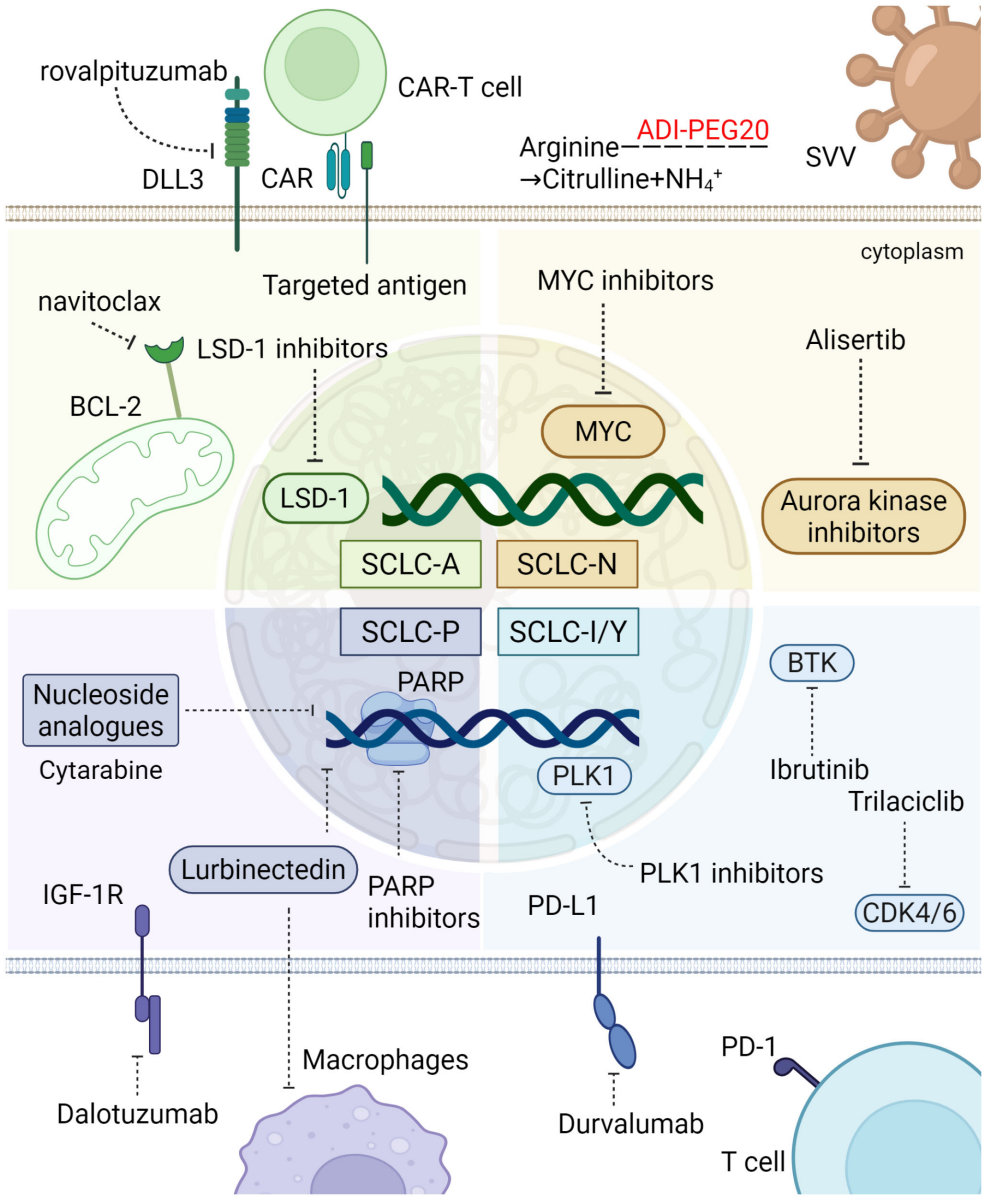
Sensitivity of different SCLC subtypes to treatment. SCLC-A, the most prevalent SCLC subtype, is marked by high NE marker expression and molecular features like elevated EZH2 and DLL3. It responds to DLL3-targeted therapies, with tarlatamab showing over 50% DCR. SCLC-N, characterized by NEUROD1 expression, is sensitive to Aurora kinase inhibitors and metabolic therapies targeting arginine metabolism. SCLC-P, with low NE markers, depends on IGF-1R and shows promise with PARP inhibitors. The inflammatory SCLC-I subtype, with high CTL and NK cell infiltration, responds well to PD-L1 blockade and may benefit from BTK inhibition and mTOR, PLK, CDK 4/6 targeted therapies. These subtypes offer specific targets for improved SCLC treatment. The figure was created with BioRender.com. Some therapeutic agents have only undergone preliminary exploration in SCLC subtypes and may emerge as potential future treatment options. The figure was created with BioRender.com.

The inflammatory SCLC-I subtype, characterized by high infiltration of CTLs, NK cells, and tumor-associated macrophages, has demonstrated heightened responsiveness to PD-L1 monoclonal antibody treatments. Retrospective studies have shown significantly better OS in patients with SCLC-I treated with chemotherapy plus atezolizumab compared to other subtypes (157). Furthermore, Bruton’s Tyrosine Kinase (BTK) is highly expressed in SCLC-I tumors and may serve as a novel therapeutic target (8). Gene expression analysis and bioinformatics studies suggest that SCLC-I and YAP1-expressing cell lines are more sensitive to inhibitors of mammalian target of rapamycin (mTOR) and Polo-like kinase (PLK) presenting additional opportunities for targeted therapies (25, 158). However, these potential targets still require further clinical validation (**Figure 5**).

### 6.5 Mixed type

**Figure 6:**
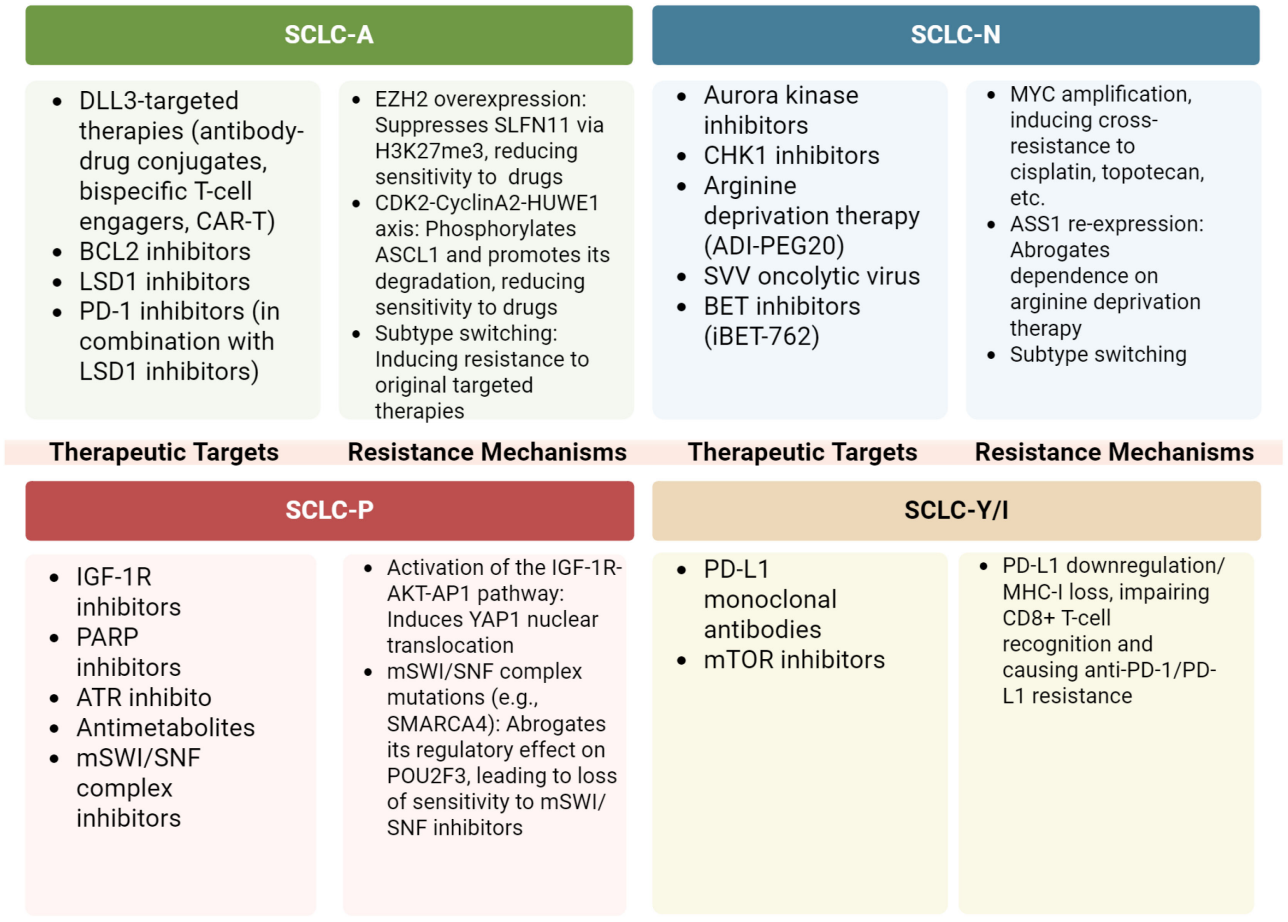
Subtype-specific vulnerabilities and resistance mechanisms. The figure was created with BioRender.com. All figures in this manuscript were created by the authors. Figures created using BioRender.com were generated under a paid subscription license, and publication rights have been obtained.

Given the intratumor heterogeneity and plasticity of SCLC subtypes, tailored treatments for patients with mixed types, particularly those with the SCLC-AN subtype, may offer more effective therapeutic outcomes. Kelenis et al. proposed that disrupting the nuclear transport of specific transcription factors by inhibiting Karyopherin subunit beta 1 (KPNB1), a nuclear transport receptor highly expressed in tumors, could represent a promising therapeutic strategy (159). Inhibitors of KPNB1, such as INI-43 and Importazole (IPZ), reduced nuclear levels of ASCL1 and NEUROD1 in several human SCLC cell lines (H2107, H2171, H524), selectively inhibiting the growth of ASCL1-positive and NEUROD1-positive SCLC cells *in vitro* and suppressing ASCL1-positive tumor growth in mice (159). Studies across multiple *in vivo* chemoresistance models have established that EZH2 promotes resistance by silencing *SLFN11* via H3K27me3, which blunts the sensitivity of tumor cells to DNA-damaging drugs (48). In addition, high EZH2 expression in the SCLC-A subtype suppresses the TGF-β–Smad–ASCL1 pathway and enhances tumor progression, indicating a promising yet unconfirmed role for EZH2 inhibitors in treating this subtype (85). While *RB1* loss abrogates the canonical CDK4/6 signaling axis, thereby conferring resistance to CDK4/6 inhibitors, evidence shows that these drugs can still counteract SCLC chemoresistance via non-canonical, *RB1*-independent mechanisms, such as through the disruption of lysosomal function and autophagy (160). Additionally, in multiple patient-derived SCLC cell lines and SCLC CDX models, Jumonji inhibitors or KDM4A knockdown led to downregulation of key markers like INSM1, ASCL1, or NEUROD1, highlighting their potential as therapeutic targets for SCLC (161). Moreover, the small molecule inhibitor iBET-762, which targets bromodomains and extra-terminal domain (BET) proteins, demonstrated selective efficacy by promoting the growth of suspended cell clusters while inhibiting the growth of adherent, mesenchymal-like cells. In SCLC PDX models, iBET-762 increased ASCL1 expression and reduced NEUROD1 and YAP1 levels, suggesting its potential for manipulating subtype dynamics in SCLC (**Figure 6**) (36).

Molecular subtype-directed precision therapy holds emerging promise for the management of SCLC. However, the clinical translation of this paradigm faces several fundamental challenges. Firstly, the inclusion of unselected patient populations in clinical trials may obscure the efficacy of targeted agents in specific molecular subsets, potentially leading to the premature abandonment of active compounds. Secondly, the intrinsic dynamic evolution of SCLC presents a major therapeutic obstacle. Intratumoral heterogeneity at diagnosis limits the representativeness of single-site biopsies, as minor, non-dominant subclones may exhibit intrinsic resistance to first-line chemotherapy or radiotherapy. When therapies are precisely designed to target the dominant subtype, they may inadvertently create a niche for the expansion of these resistant subclones, directly contributing to primary resistance and seeding future relapse (162). Under therapeutic pressure, SCLC progression and resistance are primarily driven by two mechanisms. The first is “clonal selection”: the eradication of drug-sensitive cellular populations allows pre-existing resistant subclones to proliferate, rendering treatment strategies based on initial biopsy profiles ineffective against recurrent disease—a classic manifestation of acquired resistance. The second, and more critical mechanism, is “cellular plasticity”—the ability of tumor cells to actively switch their transcriptional identity, beyond mere clonal outgrowth. For instance, under the selective pressure of a therapy targeting the SCLC-A (ASCL1-driven) subtype, cancer cells can rapidly downregulate ASCL1 and concurrently upregulate NEUROD1 or POU2F3, thereby “remodeling” their identity to evade attack and invalidating the original targeted agent. This dynamic plasticity represents a key adaptive survival strategy for SCLC and underscores the vulnerability of monotherapies targeting a single subtype. Consequently, a strategic shift in the therapeutic paradigm is imperative. Future research should prioritize the development of “combination therapies” aimed either at concurrently targeting vulnerabilities shared across multiple subtypes (e.g., DLL3) or directly attacking the core signaling pathways that drive plasticity. Furthermore, implementation of dynamic monitoring technologies such as liquid biopsy, is essential to track subtype evolution in real-time during treatment, enabling adaptive precision medicine. Ultimately, the overarching goal should be to prevent or lock tumor evolution—developing novel agents that can lock tumor cells in a drug-susceptible state or directly deprive them of their plasticity, rather than merely killing cells in their current state. Looking ahead, the systematic integration of molecular subtyping in clinical research is crucial to fully elucidate the spatiotemporal heterogeneity of SCLC before and after therapy and to objectively evaluate the effectiveness of existing regimens. This approach is a necessary pathway to overcome current limitations and ultimately achieve truly personalized treatment for SCLC patients.

## 7 Other emerging technologies

Nanotechnology demonstrates considerable potential for enhancing therapeutic outcomes in SCLC. In the realm of oral drug delivery, the encapsulation of curcumin (Cc) within natural polysaccharide-coated, lipid-based nanocarriers increased its oral bioavailability by 8.94-fold and doubled the tumor growth inhibition rate in H446 tumor-bearing mouse models (163). Furthermore, nanoformulations of irinotecan have shown clinical progress: SNB-101, a polymeric micelle containing irinotecan/SN-38, exhibited dose-dependent antitumor activity in a Phase I trial (NCT04640480) involving patients with advanced solid tumors, including SCLC (164). Meanwhile, pegylated liposomal irinotecan (Onyvide) reported an ORR of 34.5% in a Phase III trial (NCT04666648) for SCLC patients (165).

Targeting neuronal signaling pathways represents an innovative direction for SCLC therapy. Aberrant electrical activity drives SCLC malignancy via the calcium-dependent CREB/FOS pathway, providing a rationale for employing nAChR inhibitors (e.g., varenicline), calcium signaling modulators, or sodium channel blockers (55). In preclinical models, the anti-epileptic drug levetiracetam suppressed intracranial SCLC proliferation (56). While the glutamate release inhibitor riluzole monotherapy extended mouse survival to 71.5 days. Its combination with cisplatin and etoposide significantly prolonged survival by 21 days, outperforming chemotherapy alone (57). Dopamine D2 receptor agonists (cabergoline, quinpirole) not only inhibited tumor angiogenesis in PDX models but also reversed SCLC chemoresistance to cisplatin and etoposide (166).

ADCs have achieved a series of breakthroughs in SCLC treatment (167). The DLL3-targeting FZ-AD005 demonstrated potent internalization capacity and a marked bystander killing effect in preclinical models, and its clinical trial is ongoing (NCT06424665) (168). Similarly, the DLL3-targeting ZL-1310 achieved a 74% ORR in previously treated ES-SCLC patients (NCT06179069). The Trop-2-targeting sacituzumab govitecan yielded an ORR of 41.9% and a median OS of 13.6 months in the second-line setting (169). Another Trop-2-directed ADC, SHR-A1921, reported an ORR of 33.3% and a DCR of 66.7% in a Phase I trial (NCT05154604) (170). The B7-H3-targeting I-DXd demonstrated an ORR of 52.4% and a median duration of response of 5.9 months in the IDeate-PT01 trial (171). Additionally, the SEZ6-targeting ABBV-011 and CD47-blocking agents show therapeutic promise, with the latter, in combination with radiotherapy, inducing sustained antitumor immunity against SCLC in models (172, 173). Bispecific antibodies and novel immunotherapies reflect a trend towards diversified approaches. The DLL3/CD3 bispecific T-cell engager tarlatamab, combined with a PD-L1 inhibitor as maintenance therapy, demonstrated a manageable safety profile and promising antitumor activity (174). The PD-L1×VEGF-A bispecific antibody BNT327, combined with chemotherapy, achieved an unconfirmed ORR of 86.8% and a 100% DCR in treatment-naïve ES-SCLC patients (175). The novel IgG-like T-cell engager obrixtamig (BI 764532) was evaluated in a Phase I trial (NCT04429087), demonstrating an ORR of 18% and a DCR of 42% with step-up dosing in heavily pretreated patients with DLL3-positive tumors (176). Another promising agent, HPN328-4001, is being studied in a Phase I/II clinical trial to assess the DLL3/CD3 T-cell engager MK-6070 in previously treated patients with SCLC (NCT04471727). The CD3/DLL3 trispecific antibody ZG006 yielded a 66.7% ORR in early-stage trials and showed enhanced efficacy in patients with brain metastases (extracranial ORR 50% vs. 18%) (177, 178).

Combination strategies continue to be refined. Low-dose radiotherapy (15 Gy/5 fractions) combined with immunotherapy in the MATCH trial (NCT046228) achieved an ORR of 73.3% (179). Sequential therapy with camrelizumab (an anti-PD-1 antibody) plus apatinib (a VEGFR2 inhibitor) following induction chemotherapy resulted in an 88.9% ORR and a 97.2% DCR in ES-SCLC (180).

Collectively, these advances depict an innovative landscape in SCLC therapeutics, spanning from nanotechnology-enhanced drug delivery and precise intervention in neuronal signaling pathways to the development of novel targeted agents like ADCs and bispecific antibodies, alongside optimized combinations of radiotherapy, immunotherapy, and anti-angiogenic drugs. These multifaceted strategies provide a diversified arsenal for improving outcomes for SCLC patients (**Supplementary Table S1**).

## 8 Discussion

Over the past two decades, SCLC has seen progress in basic research, with clear definition of molecular subtypes (SCLC-A, SCLC-N, SCLC-P, SCLC-I), and insights into intratumoral heterogeneity, immune microenvironment, and potential therapeutic targets. However, translating these into clinical therapies faces multiple barriers. SCLC transcription factors coexist intratumorally and undergo phenotypic switching under microenvironmental regulation—e.g., chemotherapy or ASCL1-targeted therapy enriches NEUROD1/POU2F3-positive cells, causing drug resistance and recurrence. Though intermediate subtypes support evolutionary potential (66, 111), mechanisms of SCLC-P/SCLC-N switching, resistance drivers, non-NE to high-NE transition via plasticity, and links between subtype origin, spatiotemporal heterogeneity, and switching remain unclear (13, 37, 41). Dynamic subtype plasticity may select NEUROD1/POU2F3-dependent resistant clones during ASCL1-targeted therapy, leading to failure. This forces reliance on targeting pan-subtype vulnerabilities, rarely achievable due to extreme heterogeneity. In target development, SCLC differs from NSCLC (which has targetable drivers like *EGFR/ALK*). SCLC features near-complete *TP53/RB1* inactivation (tumor suppressors, not directly targetable). Research shifts to surface antigens, epigenetic regulators, and immunotherapy, but SCLC’s immunosuppressive microenvironment (cancer-associated fibroblasts, regulatory T cells forming barriers) complicates development.

Model limitations hinder translation: long-passaged cell lines lose heterogeneity; PDXs poorly mimic human immunity (especially for immunotherapy); patient-derived organoids (PDOs, supporting CD8+ T/microglia co-culture) have ~30% success for POU2F3+ subtypes (181), insufficient for pan-subtype study. Clinically, rapid progression and poor patient status limit repeated biopsies; ctDNA/CTC lacks sensitivity for recurrence monitoring (182). Unstandardized biomarkers and unselected trial populations risk misjudging drugs effective for rare subtypes (e.g., SCLC-P). Short survival, rapid recurrence, and multiple subsequent lines make demonstrating OS/PFS benefits statistically challenging. Nevertheless, advances offer hope: SCLC originates from airway basal cells with “trunk-branch” heterogeneity (basal-like trunk, NE/tuft/Atoh1+ targetable branches) (54, 183), explaining short-term efficacy followed by recurrence and guiding dynamic monitoring/trunk targeting. Bispecific antibodies in trials provide new subtype/target-specific options. Moving forward, work should focus on clarifying subtype switching and lineage plasticity to induce vulnerable phenotypes, optimizing PDOs, standardizing biomarkers, upgrading ctDNA/CTC sensitivity, and integrating biomarker screening into trial design. Multi-disciplinary collaboration may bridge the gap between basic research and clinical practice, bringing more effective treatments to patients.
