# Supplementary material for: Intratumoral heterogeneity and potential treatment strategies in small cell lung cancer
Source: Front Oncol. 2025 Oct 27;15:1657441. doi: 10.3389/fonc.2025.1657441 (PMC12597771; doi:10.3389/fonc.2025.1657441)
Supplement: Supplementary file 1 [file Table1.docx]

Supplementary Material

# Supplementary Tables

# Supplementary Table S1 : Clinical trials related to SCLC treatment*

| Treatment | Therapeutic Class | Target(s) | Drug | Trail ID | Status/Outcomes | Phase | Disease Conditions | Clinical Remark |
| --- | --- | --- | --- | --- | --- | --- | --- | --- |
| Targeted therapy | ADC | B7-H3 | HS-20093 | NCT06526624 | Not yet recruiting | 3 | LS-SCLC | • No results posted. |
|  |  |  |  | NCT06498479 | Recruiting | 3 | relapsed SCLC | • Primarily evaluate OS. |
|  |  |  |  |  |  |  |  | • No results posted. |
|  |  |  | Ifinatamab Deruxtecan | NCT06362252 | Recruiting | 1b,2 | ES-SCLC | • Primarily evaluate number of participants reporting Dose-Limiting Toxicities (DLTs) (Part 1) and overall number of participants with treatment-emergent adverse events (Part 1 and 2). |
|  |  |  |  |  |  |  |  | • No results posted. |
|  |  |  |  | NCT06203210 | Recruiting | 3 | relapsed SCLC | • Explore OS and ORR. |
|  |  |  |  |  |  |  |  | • No results posted. |
|  |  |  | ABBV-155 (Mirzo-C) | NCT03595059 | Active, not recruiting | 1 | Relapsed/refractory solid tumors | • Primarily evaluate ORR. |
|  |  |  |  |  |  |  |  | • No results posted. |
|  |  | SEZ6 | ABBV-706 | NCT05599984 | Recruiting | 1 | Advanced solid tumors | • No results posted. |
|  |  | EGFRxHER3 bispecific antibody | BMS-986507(BL-B01D1) | NCT06618287 | Not yet recruiting | 1,2a | Advanced solid tumors | • No results posted. |
|  |  | Trop-2 | Sacituzumab Govitecan(IMMU-132) | NCT03964727 | Active, not recruiting | 2 | relapsed SCLC | • Primarily evaluate ORR (part 2) and MTD (part 1).  • Show an ORR of 52.4% and a median duration of response (DOR) of 5.9 months |
|  |  |  | JS-108 | NCT04601285 | Terminated | 1 | Advanced solid tumors | • No results posted. |
|  |  |  | SKB264 | NCT04152499 | Recruiting | 1,2 | Advanced solid tumors | • Primarily evaluate ORR (part 2) and MTD (part 1). |
|  |  |  |  |  |  |  |  | • No results posted. |
|  |  | DLL3 | ZL-1310 | NCT06179069 | Recruiting | 1 | metastatic or extensive-stage SCLC | • Expore incidence of DLTs of ZL-1310 as a single agent, incidence of treatment emergent adverse-events of ZL-1310 as a single agent and so on. |
|  |  |  |  |  |  |  |  | • No results posted. |
|  |  |  | FZ-AD005 | NCT06424665 | Recruiting | 1 | Advanced solid tumors | • No results posted. |
|  |  | pH-sensitive peptide | CBX-12 | NCT04902872 | Active, not recruiting | 1,2 | Advanced or metastatic refractory solid tumors | • Phase 1 results indicated a concerning safety profile. Although preliminary antitumor activity was observed across multiple tumor types (including one case of SCLC), the objective response rate (ORR) was limited at 12.5%. |
|  |  |  |  |  |  |  |  | • No results posted. |
|  | Small molecule drug | CDK2 inhibitor | PF-07104091 | NCT04553133 | Active, not recruiting | 1,2 | advanced or metastatic SCLC | • No results posted. |
|  |  | Dual CDK4/CDK6i | Abemaciclib | NCT04010357 | Recruiting | 2 | Chemo-refractory, RB1 wild-type ES-SCLC | • Expore ORR. |
|  |  |  |  |  |  |  |  | • No results posted. |
|  |  | CDK2 degrader | NKT3964 | NCT06586957 | Recruiting | 1 | Advanced/​Metastatic Solid Tumors | • Primarily evaluate number of Participants with DLTs events. |
|  |  |  |  |  |  |  |  | • No results posted. |
|  |  | Aurora A inhibitor | Alisertib | NCT06095505 | Recruiting | 2 | ES-SCLC | • No results posted. |
|  |  |  |  | NCT02367352 | Terminated | 1b | Advanced solid tumors | • OS and PFS did not significantly improve compared to historical controls. |
|  |  |  | JAB-2485 | NCT05490472 | Recruiting | 1,2 | Advanced solid tumors | • No results posted. |
|  |  | Aurora B inhibitor | Barasertib | NCT04745689 | Active, not recruiting | 2 | ES-SCLC | • The evolving benefit-risk profile of drug did not support further development in the first-line treatment of ES-SCLC, and enrollment in study was terminated prior to study completion. |
|  |  | VEGFR2 inhibitor | Adebelimab | NCT06475209 | Recruiting | 2 | ES-SCLC | • Primarily evaluate PFS. |
|  |  |  |  |  |  |  |  | • No results posted. |
|  |  | ATR inhibitor | Berzosertib | NCT04826341 | Recruiting | 1,2 | SCLC | • No results posted. |
|  |  |  |  | NCT03896503 | Active, not recruiting | 2 | SCLC | • Combination therapy was more effective in the Platinum Response-Resistant cohort than in the Platinum Response-Sensitive cohort. |
|  |  |  | Ceralasertib | NCT04699838 | Recruiting | 2 | ES-SCLC | • No results posted. |
|  |  |  | Elimusertib | NCT04514497 | Active, not recruiting | 1 | Advanced solid tumors | • Expore the MTD (Dose Escalation Phase). |
|  |  |  |  |  |  |  |  | • No results posted. |
|  |  |  | SC0245 | NCT05731518 | Recruiting | 1b,2 | ES-SCLC | • SC0245 exhibited favorable safety and PK characteristics when administered in combination with IRI at doses ranging from 80mg QD to 120mg BID and the regimen has demonstrated preliminary antitumor activity in ES-SCLC, supporting further evaluation of the regimen in ES-SCLC and other relevant malignancies[115]. |
|  |  | WEE1 inhibitor | Debio 0123 | NCT05815160 | Recruiting | 1 | SCLC That Recurred or Progressed After Previous Standard Platinum-Based Therapy | • No results posted. |
|  |  | PERK inhibitor | HC-5404-FU | NCT04834778 | Completed | 1 | Advanced solid tumors | • Determination of MTD and DLTs of HC-5404-FU. |
|  |  |  |  |  |  |  |  | • No results posted. |
|  |  | EGFR inhibitor | HLX07 | NCT05354700 | Unknown status | 2 | ES-SCLC | • No results posted. |
|  |  | FAK inhibitor | IN10018 | NCT06030258 | Recruiting | 1,2 | ES-SCLC | • To identify the Recommended phase 2 dose of IN10018 in combination with Tislelizumab, Carboplatin and Etoposide in first-line ES-SCLC. |
|  |  |  |  |  |  |  |  | • No results posted. |
|  |  | PLK inhibitor | Onvansertib | NCT05450965 | Recruiting | 2 | Relapsed/refractory SCLC | • Primarily evaluate ORR. |
|  |  |  |  |  |  |  |  | • No results posted. |
|  |  | Pan-VEGFR inhibitor | Cediranib Maleate | NCT02899728 | Terminated | 2 | SCLC | • Inadequate accrual rate. |
|  |  |  | Lenvatinib | NCT05384015 | Recruiting | 2 | ES-SCLC | • Expore the incidence of treatment-emergent adverse events (safety and tolerability) of lenvatinib 8 mg to be used in combination with pembrolizumab plus chemotherapy (part 1) and PFS (part 2). |
|  |  |  |  |  |  |  |  | • No results posted. |
|  |  |  | Bevacizumab | NCT05588388 | Recruiting | 2 | ES-SCLC | • Expore the 6- month PFS rate. |
|  |  |  |  |  |  |  |  | • No results posted. |
|  |  | TAM receptors and VEGFR2 inhibitor | Sitravatinib | NCT05228496 | Active, not recruiting | 2 | ES-SCLC | • To assess 1-year PFS rate in maintenance phase-patient analysis set per RECIST v1.1. |
|  |  |  |  |  |  |  |  | • No results posted. |
|  |  | Pan-VEGFR and PDGFR inhibitor | Vorolanib | NCT04373369 | Terminated | 2 | ES-SCLC | • Based on the Kaplan-Meier estimate, the proportion of 6-month PFS is 27.3%. |
|  |  |  |  |  |  |  |  | • Insufficient Funding/Staff. |
|  |  | Dual PTPN2/PTPN1 phosphatase inhibitor | ABBV-CLS-484 | NCT04777994 | Recruiting | 1 | Locally Advanced or Metastatic Tumors | • No results posted. |
|  |  | Multiple kinase inhibitors | Chiauranib | NCT04830813 | Completed | 3 | SCLC | • Primarily evaluate PFS and OS. |
|  |  |  |  |  |  |  |  | • No results posted. |
|  |  |  | Surufatinib | NCT05595889 | Not yet recruiting | 2 | SCLC | • Primarily evaluate PFS. |
|  |  |  |  |  |  |  |  | • No results posted. |
|  |  |  | TT-00420 | NCT05253053 | Completed | 1,2 | Advanced solid tumors | • No results posted. |
|  |  |  | Anlotinib Hydrochloride | NCT05942508 | Recruiting | 1b | LS-SCLC | • Primarily evaluate adverse event. |
|  |  |  |  |  |  |  |  | • No results posted. |
|  |  |  |  | NCT06441344 | Not yet recruiting | 3 | ES-SCLC | • Primarily evaluate PFS and OS. |
|  |  |  |  |  |  |  |  | • No results posted. |
|  |  |  |  | NCT06611657 | Active, not recruiting | 2 | SCLC | • No results posted. |
|  |  |  | AL8326 | NCT05363280 | Active, not recruiting | 2 | SCLC | • Expore the optimal biological dose (OBD) and ORR. |
|  |  |  |  |  |  |  |  | • No results posted. |
|  |  |  | Cabozantinib | NCT04514484 | Active, not recruiting | 1 | SCLC | • Primarily evaluate the incidence of DLTs. |
|  |  |  |  |  |  |  |  | • No results posted. |
|  |  | LSD1 inhibitor | Bomedemstat | NCT05191797 | Terminated | 1,2 | SCLC | • Closed per SRC low accrual policy. |
|  |  |  | CC-90011 | NCT03850067 | Completed | 1 | ES-SCLC | • No results posted. |
|  |  |  | Iadademstat (ORY-1001) | NCT05420636 | Terminated | 2 | Relapsed/refractory SCLC | • Closed to accrual due to low probability of successful outcomes. |
|  |  | EZH2 inhibitor | PF-06821497 | NCT03460977 | Recruiting | 1 | Relapsed/refractory SCLC | • No results posted. |
|  |  |  | XNW5004 | NCT06022757 | Recruiting | 1,2 | Advanced solid tumors | • No results posted. |
|  |  | PARP1/2 inhibitor | Fluzoparib (SHR-3162) | NCT04400188 | Completed | 1,2 | Relapsed/refractory SCLC | • Until April 23, 2021, Fuzuloparib combined with SHR-1316 failed to improve the outcomes in unselected patients with relapsed SCLC. Future studies with biomarker analysis are warranted to select patients most likely to benefit from this combination treatment. Fuzuloparib 100 and 150 mg plus SHR-1316 were both tolerable with no new signals observed. |
|  |  |  |  |  |  |  |  | • No results posted. |
|  |  |  | HTMC0435 | NCT05728619 | Unknown status | 1,2 | Recurrent ES-SCLC | • No results posted. |
|  |  |  | Niraparib | NCT04701307 | Active, not recruiting | 2 | SCLC | • Primarily evaluate PFS and OS. |
|  |  |  |  |  |  |  |  | • No results posted. |
|  |  |  | Olaparib | NCT04728230 | Recruiting | 1,2 | ES-SCLC | • Expore the incidence of DLTs. |
|  |  |  |  |  |  |  |  | • No results posted. |
|  |  |  |  | NCT02734004 | Active, not recruiting | 1,2 | Advanced solid tumors | • The Duration of Response is about 3.6 months,PFS is about 2.4 months. |
|  |  |  |  |  |  |  |  | • No clear clinical benefit. |
|  |  |  |  | NCT03428607 | Completed | 2 | Relapsed SCLC | • No clear clinical benefit. |
|  |  |  | Pamiparib (BGB-290) | NCT05483543 | Unknown status | 2 | LS-SCLC | • Expore 1-year PFS. |
|  |  |  |  |  |  |  |  | • No results posted. |
|  |  |  | RP12146 | NCT05002868 | Completed | 1 | ES-SCLC | • No results posted. |
|  |  |  | Rucaparib | NCT04209595 | Completed | 1,2 | Solid tumors | • The phase II portion of the study was not done because the study was closed to enrollment due to Clovis Oncology withdrawing support for the study. |
|  |  |  |  | NCT03958045 | Completed | 2 | SCLC | • The median PFS was 11 months and the DCR is about 33.3%. |
|  |  |  | Talazoparib | NCT03672773 | Active, not recruiting | 2 | ES-SCLC | • No results posted. |
|  |  | PP2A inhibitor | LB-100 | NCT04560972 | Active, not recruiting | 1 | ES-SCLC | • Expore recommended phase 2 dose. |
|  |  |  |  |  |  |  |  | • No results posted. |
|  |  | Exportin-1 (nuclear export) inhibitor | Selinexor | NCT05975944 | Unknown status | 1,2 | ES-SCLC | • No results posted. |
|  |  | BCL2 inhibitor | APG-1252 | NCT04893759 | Terminated | 1 | Advanced Neuroendocrine Tumor | • Expore MTD determination and safety data. |
|  |  |  |  |  |  |  |  | • No results posted. |
|  |  | Vascular Disrupting Agent | Plinabulin | NCT03575793 | Completed | 1,2 | Recurrent SCLC | • The median PFS is 1.6 months and median OS is 4.2 months. |
|  |  | Topoisomerase II inhibitor | Etoposide | NCT06667167 | Not yet recruiting | 2 | ES-SCLC | • Primarily evaluate PFS. |
|  |  |  |  |  |  |  |  | • No results posted. |
|  |  | Topoisomerase I inhibitor | PLX038 (PEGylated SN38) | NCT04209595 | Completed | 1,2 | Solid tumors | • The phase II portion of the study was not done because the study was closed to enrollment due to Clovis Oncology withdrawing support for the study. |
|  |  |  |  | NCT06337630 | Recruiting | 1 | Advanced solid tumors | • No results posted. |
|  |  | CHK1 inhibitor | Prexasertib | NCT02735980 | Completed | 2 | ES-SCLC | • The median PFS is 1.4 months. |
|  | Other | Arginine ADI-PEG 20 | Arginine ADI-PEG 20 | NCT05616624 | Recruiting | 1,2 | SCLC | • No results posted. |
|  |  | GD2 | GD2-SADA:177Lu-DOTA Complex | NCT05130255 | Recruiting | 1 | GD2-expressing solid tumors | • No results posted. |
|  |  | Somatostatin receptor | [212Pb]VMT-Alpha-NET | NCT06479811 | Recruiting | 1 | Somatostatin-Receptor Positive SCLC | • Expore MTD of [212Pb]VMT-alpha-NET (dose escalation cohort) and safety of [212Pb]VMT-alpha-NET at the MTD (dose expansions cohorts). |
|  |  |  |  |  |  |  |  | • No results posted. |
| Immunotherapy | monoclonal antibodies | Anti-CTLA-4 | Ipilimumab | NCT03670056 | Active, not recruiting | 2 | ES-SCLC | • Primarily evaluate the change in the ratio of Teff/Treg cells. |
|  |  |  |  |  |  |  |  | • No results posted. |
|  |  |  | Tremelimumab | NCT03923270 | Active, not recruiting | 1 | ES-SCLC | • No results posted. |
|  |  | Anti-PD-1 | Nivolumab | NCT06646276 | Recruiting | 3 | ES-SCLC | • Primarily evaluate OS. |
|  |  |  |  |  |  |  |  | • No results posted. |
|  |  |  | Tislelizumab | NCT06536868 | Recruiting | 2 | ES-SCLC | • Primarily evaluate PFS. |
|  |  |  |  |  |  |  |  | • No results posted. |
|  |  |  |  | NCT05896059 | Recruiting | 2 | ES-SCLC | • Expore 1-year PFS rate assessed in maintenance phase-patient analysis set per RECIST v1.1. |
|  |  |  |  |  |  |  |  | • No results posted. |
|  |  |  | Toripalimab | NCT06095583 | Recruiting | 3 | LS-SCLC | • Primarily evaluate PFS and OS. |
|  |  |  |  |  |  |  |  | • No results posted. |
|  |  |  | Sintilimab | NCT05527821 | Not yet recruiting | 2 | Advanced solid tumors | • Primarily evaluate PFS. |
|  |  |  |  |  |  |  |  | • No results posted. |
|  |  |  | Serplulimab | NCT06497530 | Not yet recruiting | 2 | ES-SCLC | • Primarily evaluate PFS• No results posted. |
|  |  |  | Camrelizumab | NCT05001412 | Unknown status | 2 | ES-SCLC | • An ORR of 88.9%, a DCR of 97.2%, a median PFS of 7.3 months, and a median OS of 17.3 months. |
|  |  |  | Pembrolizumab | [NCT02402920](https://clinicaltrials.gov/ct2/show/NCT02402920) | Active, not recruiting | 1 | LS-SCLC | • No results posted. |
|  |  | Anti-PD-L1 | Atezolizumab | [NCT02763579](https://clinicaltrials.gov/ct2/show/NCT02763579) | [Completed](https://clinicaltrials.gov/ct2/show/NCT02763579) | 3 | ES-SCLC | • At a median follow-up of 13.9 months, atezolizumab significantly improved overall survival (OS) and yielded a median progression-free survival (PFS) of 5.2 months, with a 1-year OS rate of 51.7%. |
|  |  |  | Durvalumab | NCT06371482 | Recruiting | 2 | LS-SCLC | • Primarily evaluate ORR and treatment-emergent adverse events. |
|  |  |  |  |  |  |  |  | • No results posted. |
|  |  |  |  | NCT06419179 | Enrolling by invitation | 2 | SCLC | • Primarily evaluate PFS. |
|  |  |  |  |  |  |  |  | • No results posted. |
|  |  |  | Adebrelimab | NCT06614621 | Not yet recruiting | 2 | ES-SCLC | • Primarily evaluate PFS. |
|  |  |  |  |  |  |  |  | • No results posted. |
|  |  |  | Benmelstobart | NCT06469879 | Not yet recruiting | 3 | LS-SCLC | • Expore PFS. |
|  |  |  |  |  |  |  |  | • No results posted. |
|  |  | Anti BTLA | JS004 | NCT06648200 | Not yet recruiting | 2 | Relapsed and ES-SCLC | • Expore PFS. |
|  |  |  |  |  |  |  |  | • No results posted. |
|  |  | Anti LAG-3 | IBI110 | NCT05026593 | Completed | 2 | Untreated ES-SCLC | • Primarily evaluate PFS. |
|  |  |  |  |  |  |  |  | • No results posted. |
|  |  | Anti-TIGIT | Tiragolumab | NCT04256421 | Completed | 3 | Untreated ES-SCLC | • No clear clinical benefit |
|  |  |  |  |  |  |  |  | • Quality Control Review Has Not Concluded. |
|  |  | Anti-DLL3 | LB2102 | [NCT05680922](https://clinicaltrials.gov/ct2/show/NCT05680922) | Recruiting | 1 | ES-SCLC | • To characterize the safety and tolerability of LB2102 and determine recommended dose for expansion (RDE). |
|  |  |  |  |  |  |  |  | • No results posted. |
|  |  |  | Tarlatamab | NCT06598306 | Recruiting | 1b | ES-SCLC | • Primarily evaluate the number of participants with DLTs and treatment-emergent adverse events. |
|  |  |  |  |  |  |  |  | • No results posted. |
|  | Bispecific Antibody | Anti-PD-1, Anti-VEGF | AK112 | NCT05116007 | Completed | 1b | ES-SCLC | • Primarily evaluate the incidence of Grade 3 or higher adverse events and ORR. |
|  |  |  |  |  |  |  |  | • No results posted. |
|  |  | Anti-DLL3, Anti-CD47 | PT217 | NCT05652686 | Recruiting | 1,2 | Patients With Neuroendocrine Carcinomas Expressing DLL3 | • No results posted. |
|  |  | Anti-PD-L1, Anti-CD47 | IMM2520 | NCT05780307 | Recruiting | 1 | Advanced solid tumors | • No results posted. |
|  |  | Anti-DLL3, Anti-CD3 | BI 764532 | NCT06077500 | Recruiting | 1 | SCLC | • Dose escalation: the Occurrence of DLTs in the MTD evaluation period (Part 1). |
|  |  |  |  |  |  |  |  | • Dose expansion: Occurrence of DLTs during the on-treatment period (Part 2). |
|  |  |  |  |  |  |  |  | • No results posted. |
|  |  | Anti-PD-1, Anti-TGF-β | JS201 | NCT04951947 | Recruiting | 2 | SCLC | • Primarily evaluate ORR. |
|  |  |  |  |  |  |  |  | • No results posted. |
|  |  | Anti-PD-L1, Anti-TGF-β | M7824 | NCT03554473 | Completed | 1,2 | Relapsed SCLC | • No clear clinical benefit. |
|  |  | Anti-PD-L1, Anti-VEGF | BNT327 | NCT06616532 | Not yet recruiting | 3 | SCLC | • Primarily evaluate OS. |
|  |  |  |  |  |  |  |  | • No results posted. |
|  |  |  |  | NCT06449209 | Active, not recruiting | 2 | ES-SCLC | • An unconfirmed ORR (uORR) of 86.8% and a DCR of 100% were achieved, along with a favorable safety profile. |
|  |  |  |  | NCT06712355 | Active, not recruiting | 3 | ES-SCLC | • No results posted. |

*The data source from https://www.clinicaltrials.gov and the latest update date is September 23, 2025.
